# An Epithelial Integrin Regulates the Amplitude of Protective Lung Interferon Responses against Multiple Respiratory Pathogens

**DOI:** 10.1371/journal.ppat.1005804

**Published:** 2016-08-09

**Authors:** Victoria A. Meliopoulos, Lee-Ann Van de Velde, Nicholas C. Van de Velde, Erik A. Karlsson, Geoff Neale, Peter Vogel, Cliff Guy, Shalini Sharma, Susu Duan, Sherri L. Surman, Bart G. Jones, Michael D. L. Johnson, Catharine Bosio, Lisa Jolly, R. Gisli Jenkins, Julia L. Hurwitz, Jason W. Rosch, Dean Sheppard, Paul G. Thomas, Peter J. Murray, Stacey Schultz-Cherry

**Affiliations:** 1 Department of Infectious Diseases, St Jude Children’s Research Hospital, Memphis, Tennessee, United States of America; 2 Department of Immunology, St Jude Children’s Research Hospital, Memphis, Tennessee, United States of America; 3 The Hartwell Center, St Jude Children’s Research Hospital, Memphis, Tennessee, United States of America; 4 Department of Veterinary Pathology Core, St Jude Children’s Research Hospital, Memphis, Tennessee, United States of America; 5 Rocky Mountain Laboratories, NIAID, NIH, Hamilton, Montana, United States of America; 6 Division of Respiratory Medicine, University of Nottingham, Nottingham, United Kingdom; 7 Department of Medicine, Division of Pulmonary, Critical Care, Allergy, and Sleep Medicine, UCSF Medical Center, San Francisco, California, United States of America; Johns Hopkins Bloomberg School of Public Health, UNITED STATES

## Abstract

The healthy lung maintains a steady state of immune readiness to rapidly respond to injury from invaders. Integrins are important for setting the parameters of this resting state, particularly the epithelial-restricted αVβ6 integrin, which is upregulated during injury. Once expressed, αVβ6 moderates acute lung injury (ALI) through as yet undefined molecular mechanisms. We show that the upregulation of β6 during influenza infection is involved in disease pathogenesis. β6-deficient mice (β6 KO) have increased survival during influenza infection likely due to the limited viral spread into the alveolar spaces leading to reduced ALI. Although the β6 KO have morphologically normal lungs, they harbor constitutively activated lung CD11b^+^ alveolar macrophages (AM) and elevated type I IFN signaling activity, which we traced to the loss of β6-activated transforming growth factor-β (TGF-β). Administration of exogenous TGF-β to β6 KO mice leads to reduced numbers of CD11b^+^ AMs, decreased type I IFN signaling activity and loss of the protective phenotype during influenza infection. Protection extended to other respiratory pathogens such as Sendai virus and bacterial pneumonia. Our studies demonstrate that the loss of one epithelial protein, αVβ6 integrin, can alter the lung microenvironment during both homeostasis and respiratory infection leading to reduced lung injury and improved survival.

## Author Summary

The lung undergoes daily assault by microbes and other inhaled particulates and must maintain the balance between clearance of harmful microorganisms while protecting the delicate lung structure to avoid acute lung injury. Not surprisingly, this is a complex process requiring communication between the lung epithelial cells (first site of attack by invaders) and the cells of the intrinsic immune response. We demonstrate the αVβ6 integrin is an important player in this interface. Loss of αVβ6 during influenza infection, and other respiratory infections, leads to bolstered protection against severe lung disease and improved survival in mice. Even in the absence of infection, β6 KO animals have a distinct anti-microbial lung microenvironment as evidenced by increased type I IFN activity and activated alveolar macrophages that appear “poised to defend”. These studies explore how the epithelial-specific αVβ6 integrin regulates the lung microenvironment to alter alveolar macrophage activity through a TGF-β-dependent mechanism, leading to changes in both the homeostatic lung and responses to respiratory infections as a way of balancing microbial clearance with protection of the lung from excessive damage.

## Introduction

At each breath, the lung is challenged by a large number and diversity of microbes and other foreign material such as pollen and dust. Many inhaled microbes cause lethal infections if not contained by the lung immune system, which has evolved to balance rapid and efficient microbial clearance with protection of the delicate lung structure from excessive damage. Lung damage caused by microbial pathogens is the cause of acute lung injury (ALI), which leads to increased edema, alveolar permeability, and impaired oxygen exchange. In severe cases, ALI can result in impaired gas exchange function and ultimately death (acute respiratory distress syndrome or ARDS) [1]. To mitigate lung damage after infection, inflammation resolves, returning to homeostasis that restores normal lung function [2,3]. The air-interface structure of the lung involves a complex immune cell population including resident interstitial macrophages and dendritic cells, and GM-CSF-dependent alveolar macrophages [4–7]. A key question in understanding pulmonary immunity concerns how the balance between effective immune surveillance and maintenance of lung anatomy and physiology is achieved through life.

Integrins are heterodimers composed of α and β subunits that regulate a plethora of cellular functions including cell-matrix and cell-cell adhesion, cell activation, and the recognition and post-translational processing of molecules [8]. In the lung microenvironment, β6 (encoded by *Itgb6*) exclusively pairs with the αV subunit and expression of the αVβ6 heterodimer is limited to epithelial cells. In this context, αVβ6 plays a key role in controlling several steps in lung homeostasis. Mice lacking the β6 subunit (β6 KO) have highlighted the central role αVβ6 integrin plays in balancing the pulmonary environment during injury [9,10]. For example, in both bleomycin-induced and PAR1-mediated ventilator-associated lung injury and airway hyper-responsiveness models, β6 KO mice were protected from ALI [11–13]. β6 KO mice also had reduced IL-1β-mediated lung injury in a model of bronchopulmonary dysplasia due to decreased inflammation and cellular infiltrate to the lung [14]. Alveolar macrophages isolated from β6 KO are characterized as large and foamy and express high amounts of MMP12 [15,16].

Severe respiratory infections are often associated with ALI/ARDS [17–19], and the pathophysiology is similar for a variety of infectious pathogens. For example, patients with SARS and MERS-CoV were found to have increased infiltration of inflammatory macrophages, accumulation of debris in the lung, and diffuse alveolar damage [20,21]. Similarly, elderly patients suffering from RSV sustained widespread alveolar damage [22]. Bacterial pneumonia can result in increased formation of hyaline membranes [23]. Deposition of collagen and fibrin, while necessary for lung repair, can also build up and block gas exchange [24]. In the case of influenza, the major complication leading to ALI/ARDS is pulmonary edema and impaired fluid clearance due to dysregulation of transporters that clear fluid from the alveolus such as ENaC and Na^+^K^+^ATPase [23,25,26]. The virus itself can also directly kill epithelial cells [27]. ALI/ARDS has a mortality rate of 30–50%, therefore it is critical from a public health perspective to understand the mechanisms at play [28,29].

Given the role of the β6 integrin in modulating ALI, we hypothesized that its upregulation during respiratory infections such as influenza would be important for viral pathogenesis. To test this, β6 KO mice were infected with the 2009 pandemic H1N1 influenza virus. β6-deficient mice were protected from influenza virus-induced disease as well as a broad range of respiratory pathogens, in most cases independent of effects on overall microbial numbers. Mechanistically, we found that epithelial β6 controls the homeostatic lung interferon response. In the absence of β6, type I interferon signaling is constitutive, causing the host to have an advantage over the spread of the virus. This protective phenotype was reversed by exogenous TGF-β1 or elimination of the type I interferon receptor, suggesting that αVβ6 controls a communication system between lung epithelia and immune cells through a TGF-β-dependent mechanism. These studies have important implications as transient inhibition of αVβ6 may represent a potential therapy for the management of acute lung injury.

## Results

### β6 KO mice are protected from diverse respiratory infections

**Fig 1:**
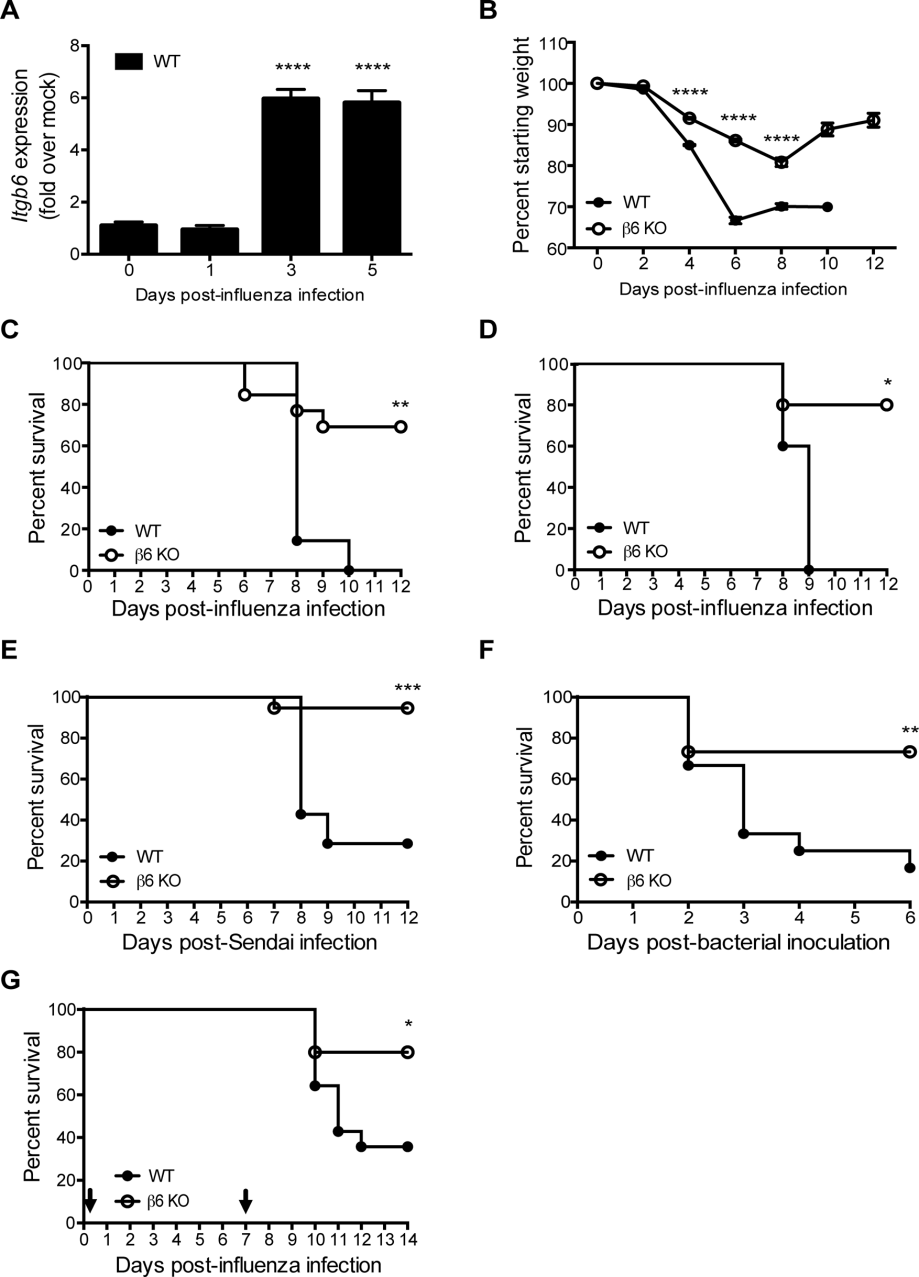
Inhibiting increased β6 integrin levels during respiratory infections is protective. β6 KO or WT mice were intranasally inoculated with CA/09 H1N1 influenza virus (10^4^ TCID_50_) and monitored for (A) β6 mRNA expression in lungs as quantitated by qPCR and normalized to GAPDH as a control. Data is pooled from 2 independent experiments with n = 2 mice per group. p< 0.0001 by Student’s *t* test; (B) weight loss and (C) survival for 12 dpi. Data from (B) is pooled from 6 independent experiments with n = 4–12 mice per group. (C) is pooled from 2 independent experiments with n = 6–7 mice per group. Error bars indicate SEM. p < 0.0001 by two-way ANOVA with Bonferroni post-test (B) or p < 0.01 by log-rank (Mantel-Cox) test (C). (D) Mice were intranasally inoculated with 10^4^ TCID_50_ of A/Anhui/1/2013 H7N9 influenza virus and monitored for survival for 12 dpi. Data represents a single experiment with n = 5 mice per group. p < 0.05 by log rank (Mantel-Cox) test. (E) Mice were intranasally inoculated with 2.0 x 10^4^ plaque forming units (PFU) Sendai virus and monitored for morbidity and mortality for 12 dpi. Data is pooled from 2 independent experiments with n = 5–10 mice per group. p < 0.001 by log rank (Mantel-Cox) test. (F) Mice were inoculated with 5X the LD_100_ of *S*. *pneumoniae* (D39X) and monitored for survival. Data is pooled from 3 independent experiments with n = 3–5 mice per group. p < 0.01 log rank (Mantel-Cox) test. (G) Mice were inoculated with 80 TCID_50_ of A/Puerto Rico/8/34 influenza virus followed by inoculation with 100 CFU of *S*. *pneumoniae* (D39X) at 7 days post-influenza infection and monitored for survival. Data is pooled from 3 independent experiments with n = 3–5 mice per group. p < 0.05 log rank (Mantel-Cox) test.

β6 expression is induced upon mechanical or inflammatory injury and is an important mediator of ALI [10,12,14,30]. Thus, we hypothesized that upregulation of the β6 integrin during respiratory infections would be involved in viral pathogenesis. To test this, we measured β6 expression by quantitative RT-PCR at different times post-influenza infection. Consistent with lung injury models, β6 mRNA was significantly upregulated in whole lung homogenates by 3 days post-infection (dpi) and expression remained elevated through 5 dpi (p < 0.0001) correlating with the appearance of ALI (Fig 1A). We then intranasally infected WT and *Itgb6*-deficient mice (β6 KO mice) with A/California/04/2009 (CA/09) H1N1 virus and monitored morbidity for 12 dpi. Compared to WT controls, β6 KO mice lost significantly less weight (4, 6, and 8 dpi p < 0.0001) and began to recover by 10 dpi, while WT mice lost weight and either succumbed to infection or were euthanized due to morbidity between 6–10 dpi (Fig 1B and 1C). The dose of CA/09 virus used (10^4^ TCID_50_) was lethal to all WT mice, while 70% β6 KO mice survived the infection (p = 0.0090). Mouse lethal dose 50 (MLD_50_) studies demonstrated the resistance of the β6 KO to influenza infection, with WT mice having an MLD_50_ of 10^2.5^ versus 10^5.3^ for the KO to the CA/09 virus. Protection was not limited to H1N1 infection; β6 KO mice were also protected from an emerging strain of avian influenza virus associated with severe and even fatal human respiratory disease [17], A/Anhui/1/2013 (H7N9) influenza virus (p = 0.0326, Fig 1D). β6 KO mice were also significantly protected from a lethal challenge of Sendai virus (p = 0.006, Fig 1E) and *Streptococcus pneumonia* (p = 0.0082, Fig 1F).

A major risk factor for influenza infection is secondary bacterial pneumonia [31]. As β6 KO mice were protected from individual influenza and *S*. *pneumoniae* challenges, we tested the β6 KO mice with a secondary bacterial challenge model. Mice were inoculated intranasally with a sublethal dose of A/Puerto Rico/8/34 H1N1 influenza virus, and 7 dpi administered a low dose of *S*. *pneumoniae* (D39X strain) [32]. This dual infection model is generally lethal in WT mice, although neither challenge alone causes death [33]. Survival of WT mice after secondary challenge was ~30%, while β6 KO mice were significantly protected (p = 0.0226) with 70% survival (Fig 1G) highlighting the importance of β6 integrin in the pathogenesis of respiratory infections.

### β6 KO mice have decreased ALI and influenza spread within the lungs

**Fig 2:**
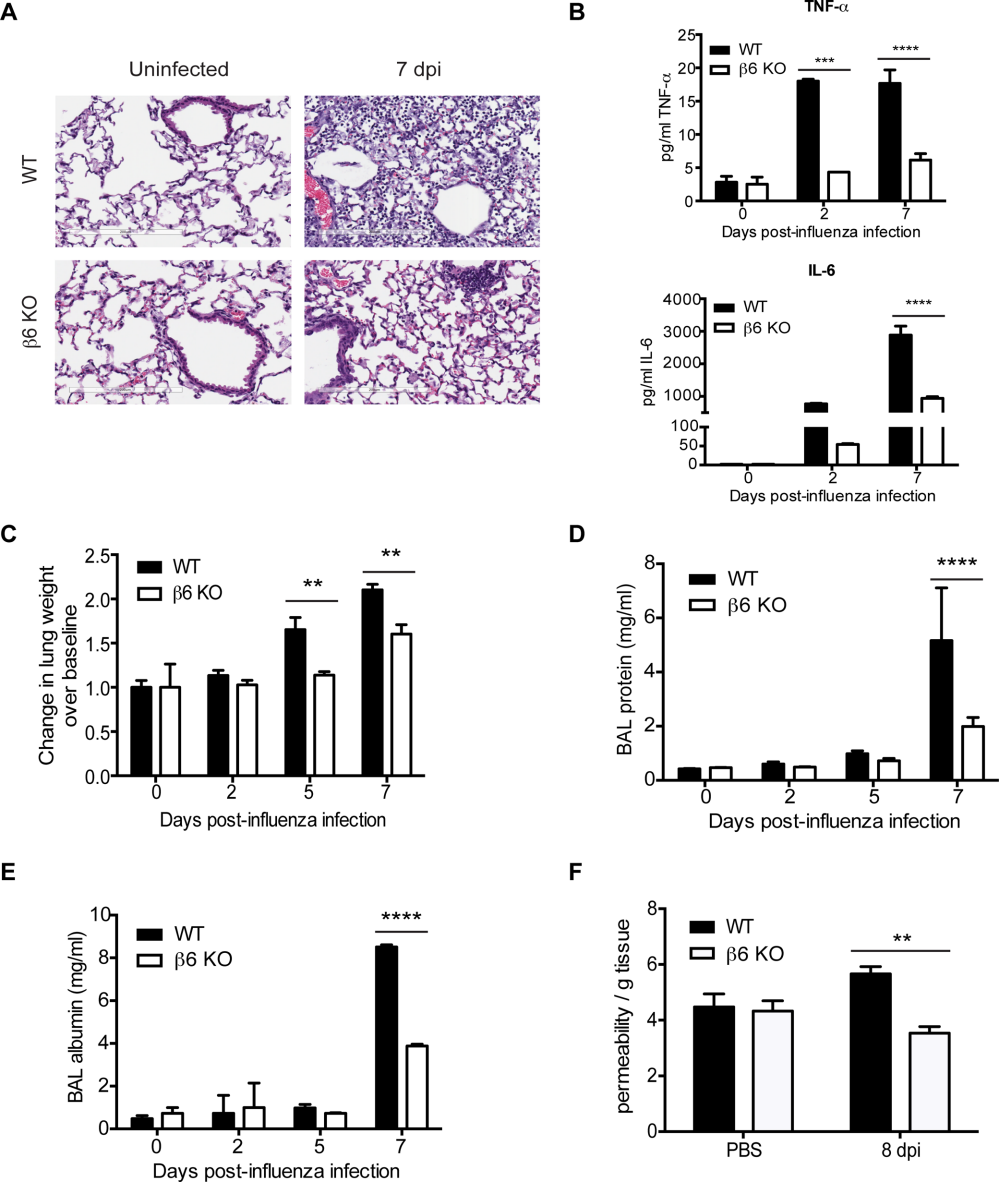
Lack of β6 correlates with improved lung permeability during influenza virus infection. (A) Whole lung sections from CA/09 H1N1 influenza virus inoculated mice as described in Fig 1 were stained by H&E. Data are representative from 6 independent experiments with n = 2–4 mice per group. Bar = 200 μm. (B) TNF-α (upper panel) and IL-6 (lower panel) were quantified from the BALF of infected mice by ELISA. (C) Lungs from CA/09 H1N1 influenza virus inoculated mice sacrificed at 2, 5, and 7 dpi were harvested and immediately weighed then dried at 60°C for 96 h before obtaining dry weights. Wet weight was normalized to dry weight of each individual lung before calculating fold increase over mice inoculated with PBS (uninfected). Data are pooled from 2 independent experiments with n = 2–3 mice per group. Error bars indicate SEM. p < 0.01 at 5 and 7 dpi by two-way ANOVA with Bonferroni post-test. (D) and (E) At 2, 5, and 7 dpi, mice were sacrificed, BALF collected, total protein levels measured by BCA assay (D) and albumin levels measured by ELISA (E). Data are from 6 independent experiments with n = 2–4 mice per group run in triplicate. Error bars indicate SEM. p < 0.0001 at 7 dpi by two-way ANOVA with Bonferroni post-test. (F) At 8 dpi, mice were i.v. injected with 20 mg/kg Evans blue dye 2 h before sacrifice. Endothelial permeability of the lung was measured by quantification of the amount of dye retained by the tissue. Data was normalized to the dry weight of the lung tissue. Data are pooled from 2 independent experiments with n = 3–4 mice per group run in triplicate. Error bars indicate SEM. p < 0.01 by Student’s *t* test.

To determine if the enhanced survival seen in the β6 KO mice was associated with decreased ALI, we examined histological sections for evidence of tissue injury, alterations of the alveolar capillary barrier, and inflammatory responses [1]. Although groups were histologically similar at 3 dpi, at 7 dpi inflammation and thickened septa involving extensive areas of alveolar parenchyma was seen in WT lungs. In marked contrast, evidence of inflammation and tissue damage in the β6 KO lungs was generally limited to terminal bronchioles and small numbers of adjacent alveoli (Fig 2A). Infected WT mice had increased TNF-α and IL-6 throughout the duration of infection, while the inflammatory cytokine response in β6 KO mice was less robust (Fig 2B). Evidence of alterations to the alveolar capillary barrier was found in infected WT mice beginning at 5 dpi as compared to KO mice (Fig 2C–2F) including heavier lungs as determined by wet/dry weight ratios (5 dpi p = 0.0077, 7 dpi p = 0.0063) indicating increased edema (Fig 2C), accompanied by increased total protein (p < 0.0001, Fig 2D) and albumin in the bronchoalveolar lavage fluid (BALF) (7 dpi p < 0.0001, Fig 2E) and accumulation of Evans blue dye in the lungs of WT mice (p = 0.0071, Fig 2F). Overall, β6 KO had decreased inflammation, acute lung injury, and improved survival during influenza infection.

**Fig 3:**
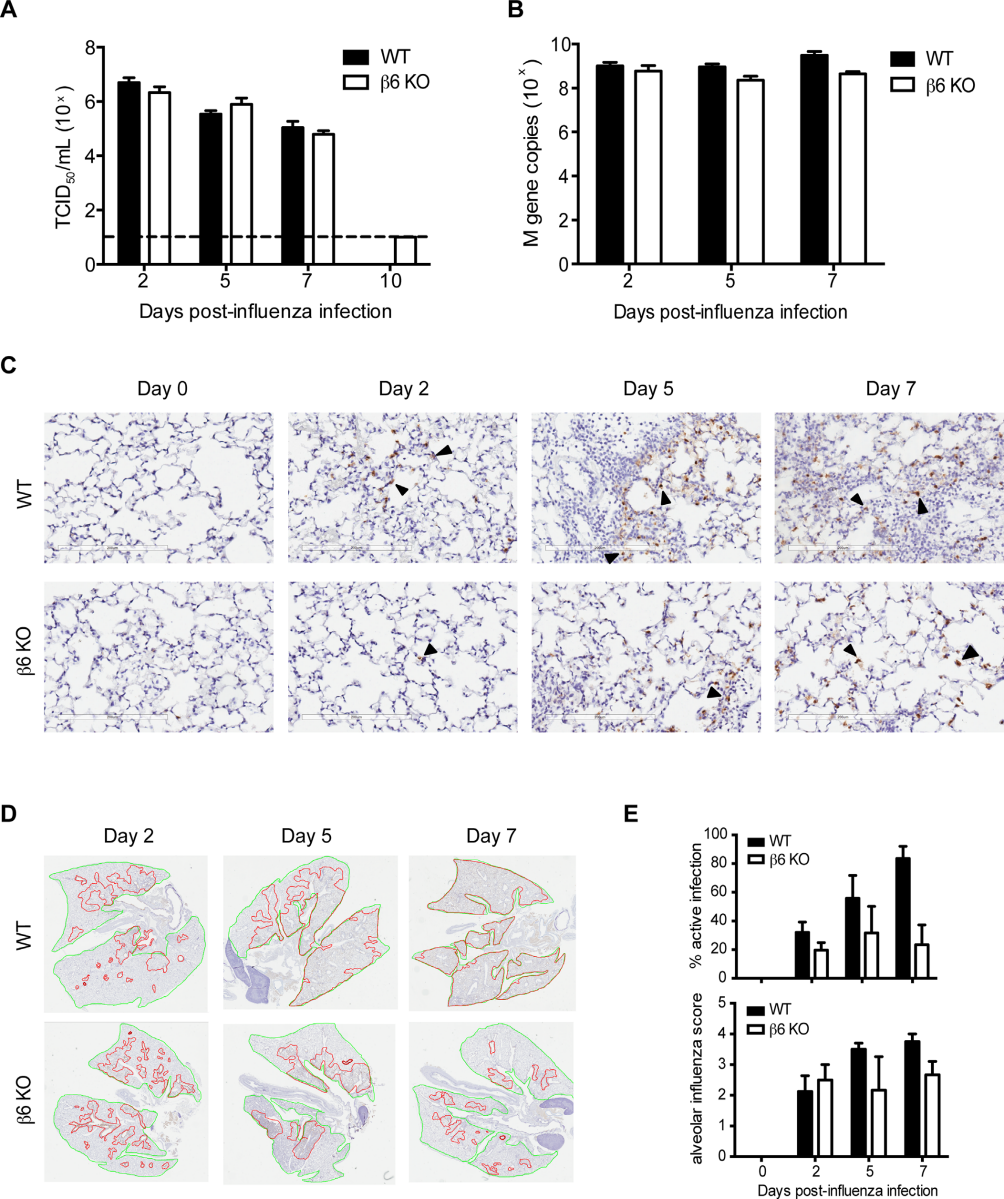
Improved survival in β6 KO mice is independent of overall viral titers, although lung viral spread is affected. (A) Viral titers in whole lung homogenates from CA/09 influenza were determined by TCID_50_ assay. Dotted line = limit of detection. Data are from 6 independent experiments with n = 3–6 mice per group run in triplicate. Error bars indicate SEM. Not statistically significant by two-way ANOVA with Bonferroni post-test. (B) RNA was extracted from the lungs of infected mice and viral replication was determined by real time PCR assay targeting the influenza M gene. Data are representative of 6 independent experiments with n = 3–4 mice per group run in triplicate. Error bars indicate SEM. Not statistically significant by two-way ANOVA with Bonferroni post-test. (C) Whole lung sections were paraffin-embedded and stained for influenza NP antigen at 2, 5, and 7 dpi. Bar = 200 μm. (D) Map of WT or β6 KO lungs showing sites of active infection (red) and areas of inactive infection or repair (green). Data is representative of 2 independent experiments with n = 2–4 mice per group. (E) Percentage of lung with sites of active infection (top panel) and alveolar influenza score (lower panel). The distribution of influenza was scored as follows: 0 = No virus positive cells; 1 = Small numbers of virus antigen positive cells in a few bronchioles. Scattered virus positive macrophages and alveolar type II pneumocytes mainly restricted to peribronchiolar alveoli; 2 = Multifocal virus antigen positive bronchioles, often with less than half epithelial cells infected. Numerous virus positive macrophages and type II pneumocytes restricted to peribronchiolar alveoli; 3 = Numerous virus antigen positive bronchioles. Virus positive cells are common in peribronchiolar alveoli and extend into surrounding parenchyma; 4 = Most bronchioles and majority of cells in bronchioles are antigen-positive. Majority of alveoli contain antigen positive alveolar macrophages and type II pneumocytes.

The most likely mechanism of protection in influenza infected β6 KO mice was decreased viral titers. However, tissue culture infectious dose 50 (TCID_50_) and quantitative real-time PCR showed no significant differences in viral titers, although RT-PCR suggested a trend towards lower titers in β6 KO mice (Fig 3A and 3B). We therefore spatially monitored viral spread in the lungs by immunohistochemistry for viral nucleoprotein (NP) and use of a reporter virus [34,35]. Viral spread into the alveolar spaces was reduced in β6 KO mice in comparison to WT mice, which had extensive NP staining of type II pneumocytes and macrophages in the alveolar spaces by 5 dpi. In contrast, NP staining in β6 KO mice was largely restricted to the terminal bronchiolar epithelium and adjacent alveoli (Fig 3C). This is even more evident using our NLuc CA/09 reporter virus, which clearly demonstrates decreased virus in β6 KO ([34,35], S1 Fig). Finally, WT lungs had a higher percentage of sites of active infection at 5 and 7 dpi as well as more viral antigen detected in the alveolar spaces (Fig 3D and 3E). Collectively, viral spread in KO lungs was limited compared to WT mice, with less extensive involvement of alveolar epithelial cells possibly explaining the improved lung function relative to controls. These findings are consistent with studies suggesting that increased survival during influenza infection can be independent of changes in viral titers [36–40].

### Loss of the β6 integrin leads to a modified lung microenvironment

**Fig 4:**
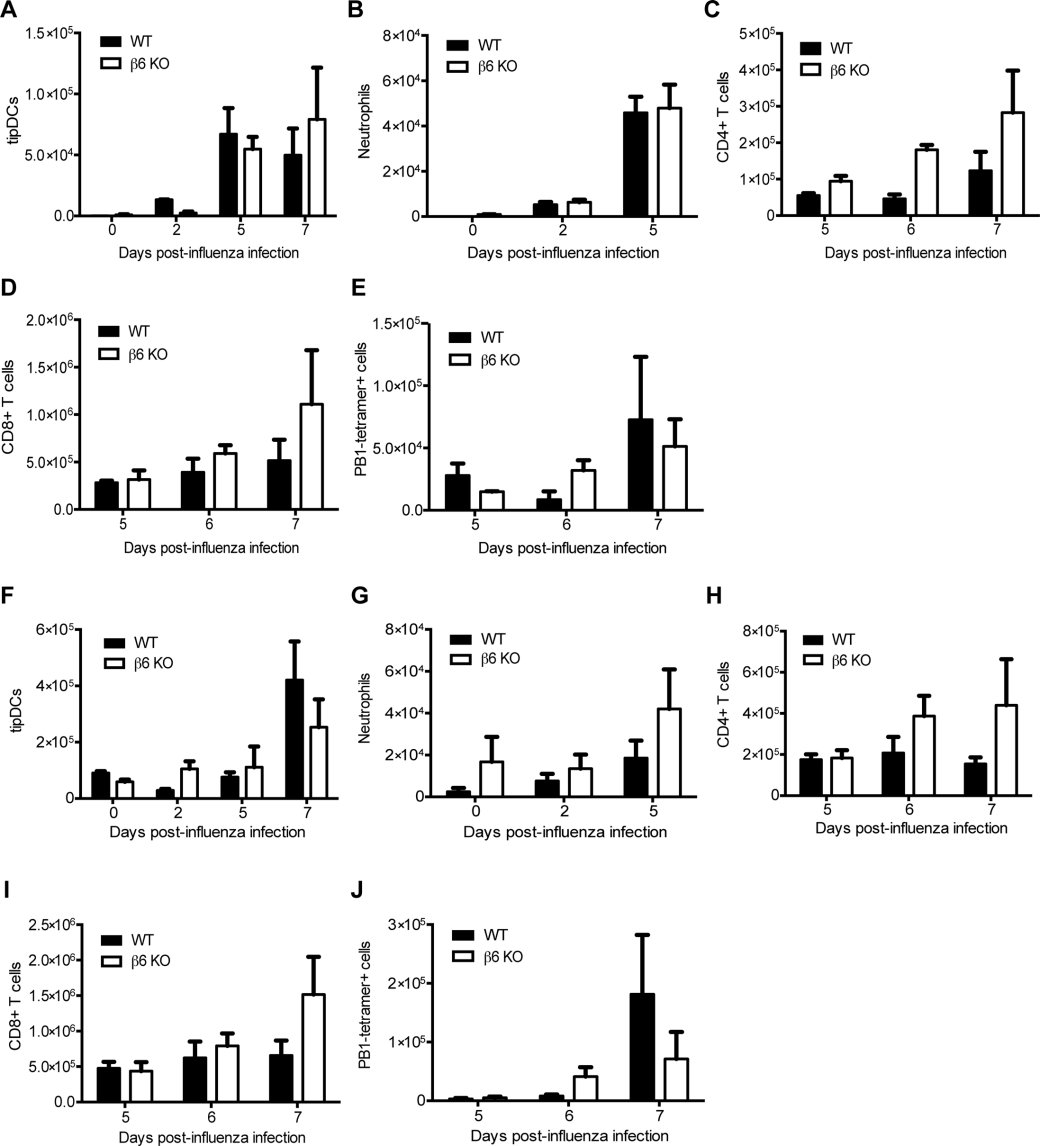
Immune cell populations in the BALF and lung. WT and β6 KO mice were infected with influenza as described in Fig 1 and BALF (A–E) or whole lung (F–J) collected at the indicated times post-infection. Numbers of tipDCs (A, F), neutrophils (B, G), CD4^+^ T cells (C, H), CD8^+^ T cells (D, I), and PB1-tetramer^+^ CD8^+^ T cells (E, J) were quantified. Data is pooled from 2 independent experiments, n = 3–5 mice per group. No significant differences were noted.

**Fig 5:**
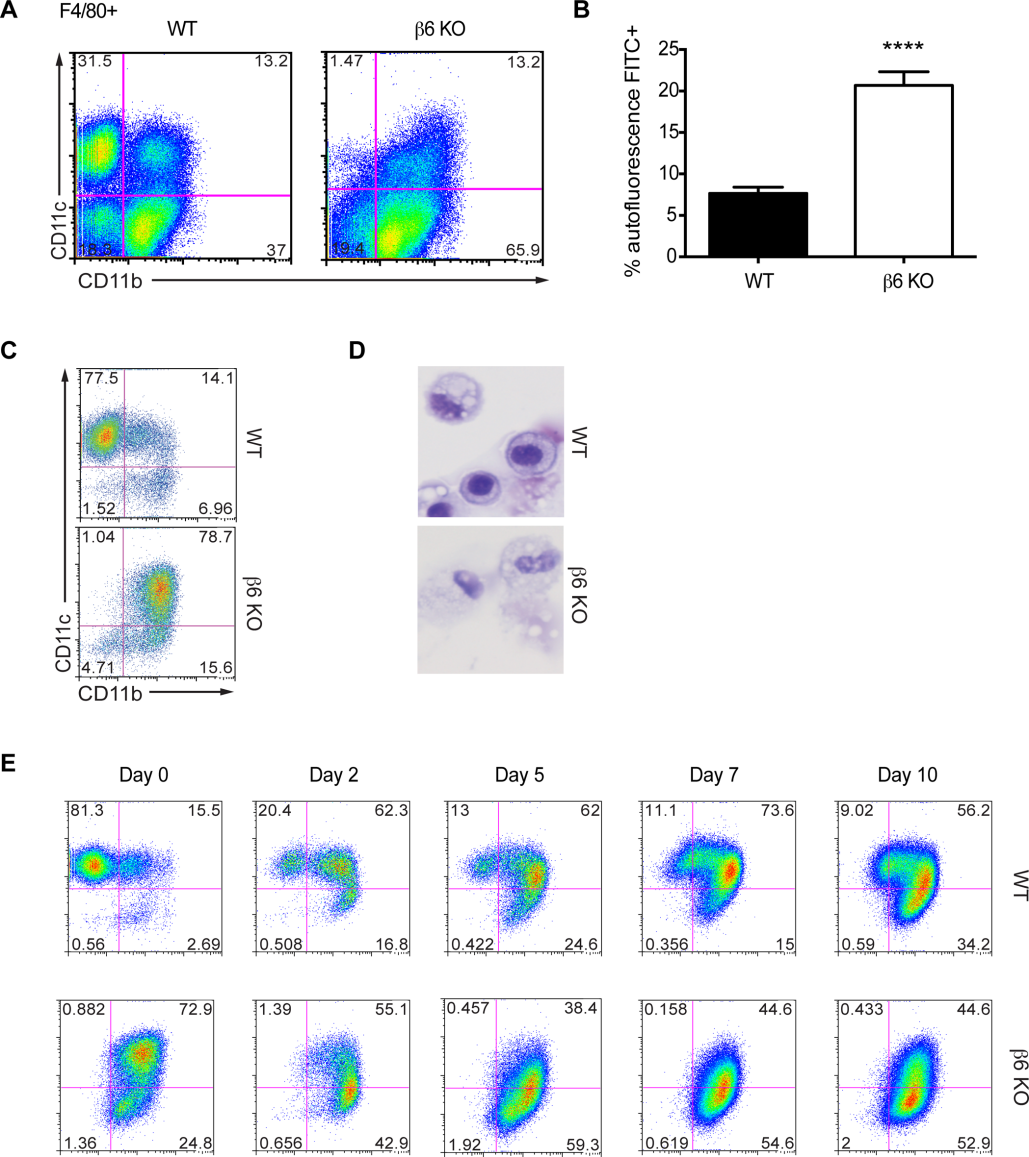
Altered alveolar macrophage phenotype in the lungs of β6 KO mice. (A) Cells were isolated from the lungs of uninfected WT or β6 KO mice and gated on F4/80^+^ cells then divided into macrophage and DC populations by CD11b and CD11c expression. (B) Quantification of the percentage of autofluorescent F4/80^+^CD11c^+^CD11b^+^ cells. p < 0.0001 by Student’s *t* test. (C) Macrophages were isolated from the lungs of uninfected WT (littermate controls) and β6 KO mice using the gating strategy shown in S2A Fig. (D) Cytospin analysis was performed on sorted macrophages representing the dominant populations of WT or β6 KO mice. (E) Macrophage populations were analyzed from the digested lungs of WT and β6 KO mice at the indicated times post-influenza inoculation. Data is representative of two independent experiments, n = 3–5 mice per group.

To understand the underlying mechanism(s) for the decreased viral spread within the lungs of β6 KO mice, we performed flow cytometry on BALF and lungs at different times post-infection. No significant differences were noted in numbers of TNF-α/iNOS-producing (tip)DCs, neutrophils, CD4^+^ T cells, CD8^+^ T cells, or influenza PB1-specific CD8^+^ T cells in either BALF (Fig 4A–4E) or whole lung (Fig 4F–4J). However, we noted substantial differences in the resident lung F480^+^ CD11c^+^ CD11b^+^ populations (macrophages and dendritic cells), namely that β6 KO mice lacked a conventional CD11c^+^ alveolar macrophage (AM) population (Fig 5A). AMs have characteristic flow cytometry properties as compared to dendritic cells (DCs) or incoming activated bone marrow-derived inflammatory monocytes including autofluoresence in the FITC channel, high constitutive expression of CD11c, F4/80 and SiglecF and low expression of CD11b [4,41,42]. By contrast, incoming activated bone marrow-derived inflammatory monocytes express CD11b, providing a means to distinguish between resident AM, inflammatory bone marrow-derived monocytes and DCs [4,43]. When we examined the lung macrophage/DC populations in uninfected β6 KO mice we found they had higher numbers of autofluorescent cells indicating AMs (Fig 5B) but instead of the normal CD11c^hi^ CD11b^-^ phenotype, the vast majority of the β6 KO AM were MerTK^+^, CD64^+^, CD11c^+^, and uniformly CD11b^+^ (Fig 5C; See S2 and S3 Figs for gating strategies). These cells maintained expression of F4/80 and SiglecF, suggesting they are AMs with altered properties. They were also phenotypically distinct from those of WT mice, with a ‘foamy’ appearance typical of activated macrophages (Fig 5D). Importantly, the β6 KO CD11c^+^ CD11b^+^ AM were present at the earliest times tested (d3 after birth), continued to be present at all points of adulthood (S4A Fig), and were not affected by reducing neonatal exposure to airborne particulate matter by using HEPA-filtered cages or low dust cage bedding (S4B Fig). Even throughout the course of influenza infection very few CD11c^hi^ CD11b^-^ cells were present in the lungs of β6 KO mice (Fig 5E). As the lungs from β6 KO were overtly normal, we concluded the CD11c^+^ CD11b^+^ cells were sufficient to perform the normal activities of lung homeostasis [12,14,16,30].

Most pulmonary AM originate from embryonic erythro-myeloid progenitors whose replacement by blood monocytes under steady state is extremely slow [42,44]. To test whether the β6 KO CD11c^+^ CD11b^+^ AM were predominantly derived from embryonic progenitors, we created mice where the blood monocyte pool was severely depleted by loss of the chemokine receptor CCR2, which is the predominant mediator for monocyte egress from the bone marrow. In the lungs of β6/CCR2 doubly-deficient mice, CD11c^+^ CD11b^+^ AM were present in identical amounts compared with those of β6 KO mice, arguing these cells are unlikely to be derived from peripheral blood monocytes (S4C Fig). Although other unknown pathways to recruit monocytes to the lung cannot be discounted, they are not as well characterized or understood as the CCR2 pathway. Taken together, we concluded loss of an epithelial integrin alters the phenotype of lung AMs.

**Fig 6:**
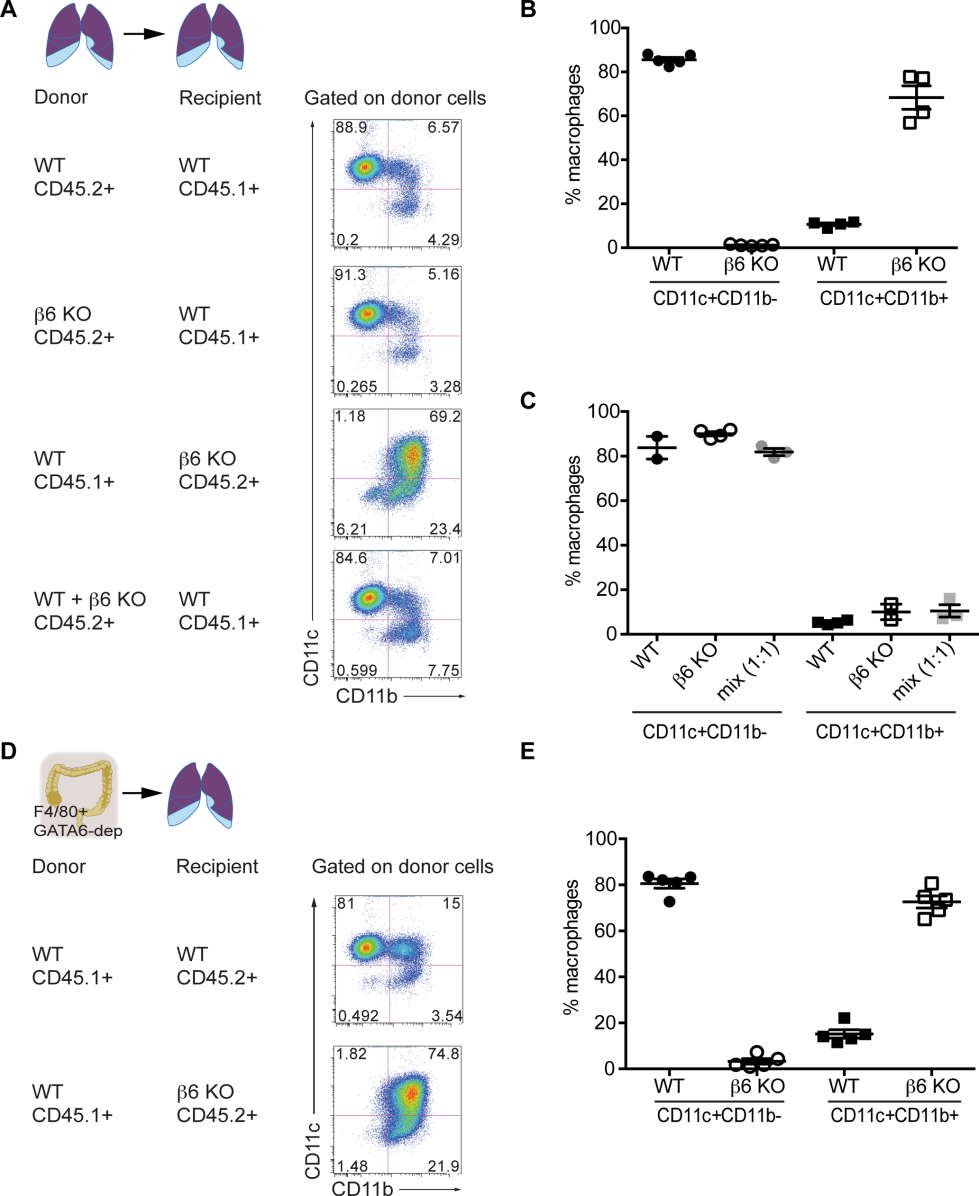
Macrophage phenotype is defined by the pulmonary microenvironment. (A) Uninfected WT (littermate controls) or β6 KO mice were inoculated intratracheally with alveolar macrophages (AM) isolated from WT or β6 KO mice. CD45.1 or CD45.2 markers identified donors and recipients. At 7 days post-transfer, animals were sacrificed and alveolar macrophages were analyzed. (B) Quantitation of macrophages transferred from WT or β6 KO mice (CD45.2) into WT CD45.1 mice. Error bars represent SEM. (C) Quantitation of macrophages transferred from WT CD45.1 mice into WT or β6 KO mice (CD45.2). Error bars indicate SEM. (D) Uninfected WT (littermate controls) or β6 KO mice were inoculated intratracheally with peritoneal macrophages (PDM) isolated from WT mice. CD45.1 or CD45.2 markers identified donors and recipients. At 7 days post-transfer, animals were sacrificed and alveolar macrophages were analyzed. (E) Quantitation of macrophages in Fig 6D. Error bars indicate SEM. Data is pooled from 2 independent experiments with n = 3–5 mice per group.

Next we tested whether the β6 KO CD11c^+^ CD11b^+^ AM were a consequence of exposure to an altered lung microenvironment caused by loss of β6, or were caused by a cell-intrinsic mechanism. Adoptive transfer of congenically marked WT AM into β6 KO animals led to the CD11c^+^ CD11b^+^ AM phenotype after 7 days while transfer of β6 KO AMs into WT animals results in a WT CD11c^hi^ CD11b^-^ phenotype (Fig 6A–6C). Importantly, the effect of the β6 KO lung microenvironment was not unique to transferred AM. Adoptive transfer of peritoneal resident macrophages into β6 KO animals resulted in conversion into the CD11c^+^ CD11b^+^ AM phenotype (Fig 6D and 6E) highlighting that it is the altered lung microenvironment of the β6 KO that controls AM phenotype. Donor cell recovery was consistent for all experiments (S5 Fig).

**Fig 7:**
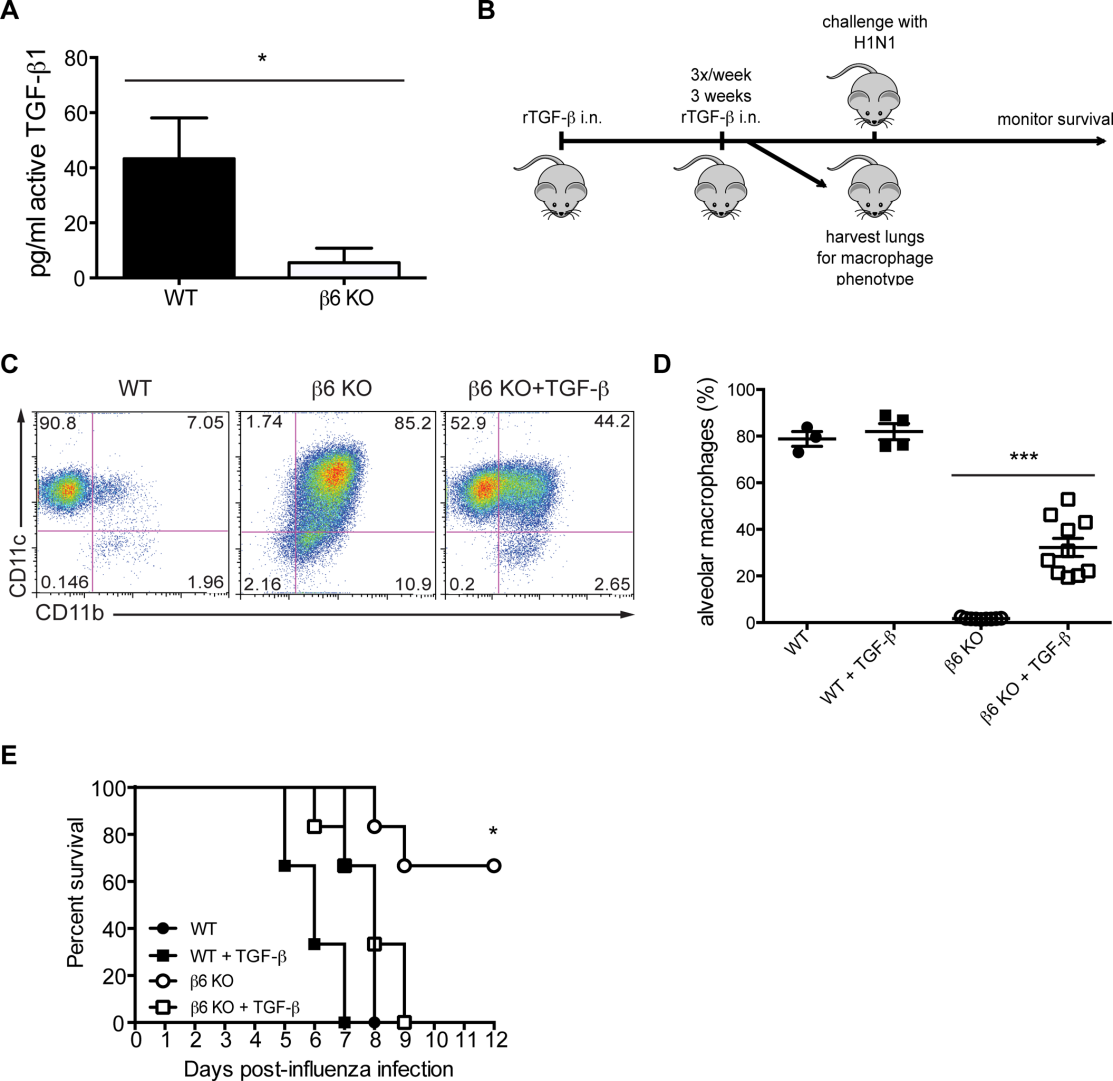
Exogenous TGF-β1 reverses macrophage phenotype and reverses protection from influenza infection. (A) Active TGF-β1 was quantified by ELISA in WT (Jackson labs) or β6 KO mice at homeostasis. (B) Experimental design describing the treatment of mice with exogenous TGF-β1. (C) Macrophages were isolated from the lungs of uninfected WT or β6 KO mice treated with or without TGF-β1 using the gating strategy shown in S2A Fig. Data reflects 2 independent experiments with n = 2–6 mice per group. (D) Quantification of CD11c^+^CD11b^-^ macrophages in mice treated with exogenous TGF-β1. Data reflects 2 independent experiments with n = 2–6 mice per group. ****p < 0.0001 and ***p < 0.001 by one-way ANOVA with Bonferroni post-test. Error bars indicate SEM. (E) Survival of influenza virus infected β6 KO or WT mice treated with TGF-β1. Data are pooled from 2 independent experiments with n = 2–6 mice per group. β6 KO mice vs β6 KO mice + TGF-β1 p < 0.05 by log-rank (Mantel-Cox) test.

What could be different about the β6 KO lung microenvironment? β6 integrin is the primary way to endogenously activate transforming growth factor-β (TGF-β) in the lung, which is known to restrain AM activity [16,45]. Thus, we next asked if the altered macrophage phenotype in the β6 KO mice was TGF-β1 dependent. As expected, endogenous lung TGF-β1 activity was reduced in β6 KO mice as compared to WT (Fig 7A). To test the hypothesis that reduced TGF-β1 activity contributed to the altered lung microenvironment of β6 KO mice, we intranasally administered exogenous TGF-β1 to WT and β6 KO mice every other day for 3 weeks (Fig 7B) as described [46]. β6 KO mice treated with TGF-β1 had increased numbers of CD11c^+^CD11b^-^ macrophages (Fig 7C, quantification Fig 7D), partially reversing the CD11c^+^CD11b^+^ phenotype, and were no longer protected from influenza infection (Fig 7E) suggesting that the loss of β6-mediated TGF-β1activation is important for both protection and the ‘activated’ macrophage phenotype.

### β6 KO mice have increased Type I IFN signaling

**Fig 8:**
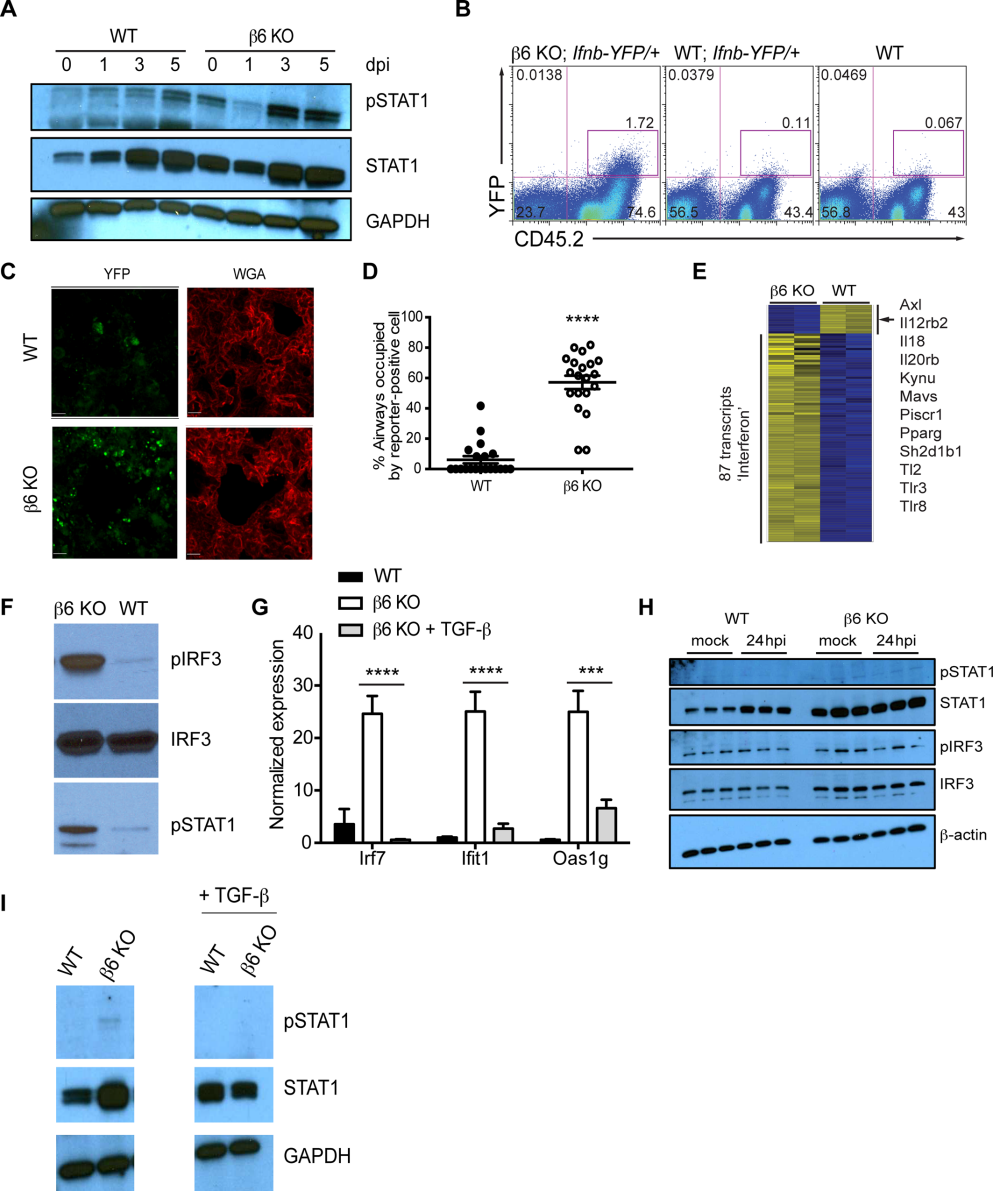
β6 KO mice have increased type I IFN activation. (A) Whole lung homogenates from WT or β6 KO mice were probed for pSTAT1 and total STAT1. GAPDH was used as a loading control. (B) β6 KO were crossed to an IFN-β-YFP reporter. Staining for YFP and CD45 is shown. (C) Lung sections of uninfected WT or β6 KO YFP reporter mice were analyzed for YFP expression (green). Cells were counterstained with WGA (red). (D) Quantification of YFP^+^ cells in WT versus β6 KO lung sections. Ten fields were imaged for each mouse. p < 0.0001 by t test. (E) Heat map of selected interferon-regulated genes from sorted WT autofluorescent CD11c^+^CD11b^-^ AM and isolated β6 KO autofluorescent CD11c^+^CD11b^+^ ‘AM’ from two independent experiments (yellow indicates higher expression; blue indicates lower expression) by z-score from -1.5 to +1.5. (F) Western blot analysis of lysates from sorted alveolar macrophage populations were probed for pIRF3, total IRF3 used as a loading control, and pSTAT1. (G) RNA was isolated from macrophage populations described in (E) and expression of interferon-related transcripts Irf7, Ifit1, and Oas1g determined by quantitative real-time PCR. ****p < 0.0001 and *** p < 0.001 by two-way ANOVA with Bonferroni post-test. Error bars indicate SEM. (H) Immunoblot analysis of primary mTECs at 24 hpi compared to uninfected. Cells were grown to a confluent monolayer and infected with CA/09 virus (MOI 0.1). Membranes were probed for phosphorylated and total STAT1 or IRF3. β-actin was used as a loading control. Data is representative of 2 independent experiments with n = 2–3 samples probed. (I) Immunoblot analysis of whole lung homogenates from WT and β6 KO were probed for phosphorylated STAT1 and total STAT1. GAPDH was used as a loading control. Data is representative of 2 independent experiments with n = 4–5 samples probed.

β6 KO mice had an altered lung environment resulting in decreased viral spread. Type I IFNs are amongst the most potent cytokines limiting viral spread. Thus, we tested the hypothesis that β6 KO mice had increased type I IFN than WT. We used phosphorylated STAT1 levels (pSTAT1) as a marker for type I IFN signaling. Increased pSTAT1 along with increased type I IFN gene expression and unchanged expression of type II IFN-dependent targets such as IDO1 are indicative of a type I IFN dominated environment. Further, STAT1 is a robust marker of type I IFN signaling due to its weak activation by other cytokines such as IL-6 [47]. Immunoblot analysis of whole lung homogenates showed the β6 KO mice had robust type I IFN signaling at baseline that increased 3–5 dpi (Fig 8A). We sought to determine the source of type I IFNs and how epithelial-expressed β6 regulated the IFN response. We noted that type I IFN mRNAs were not substantially altered in uninfected β6 KO mice (S6A–S6D Figs). Furthermore, ELISA measurements for type I IFNs did not show substantial differences between WT and β6 KO mice within whole lung homogenates. Therefore, we generated β6 KO mice crossed to an IFN-β-YFP reporter strain. In these mice, YFP expression in the whole lung was predominantly found in CD45^+^ cells and increased in the absence of β6 (Fig 8B); although immunofluorescent microscopy of frozen lung sections from WT and β6 KO YFP reporter mice showed increased YFP^+^ cells scattered throughout the lung; including in the epithelia (Fig 8C and 8D). Further delineation of the exact cell types in this system expressing YFP is precluded by the strong autofluorescence in the FITC channel in lung macrophages [4]. Thus, we examined isolated AMs and primary epithelial cells grown at the air-liquid interface from β6 KO and WT mice for increased type I IFN signaling.

Sorting autofluorescent CD11c^+^CD11b^-^ AM from uninfected WT mice and comparing them by microarray to autofluorescent CD11c^+^CD11b^+^ ‘AM’ from β6 KO demonstrated that β6 KO AM showed a striking enrichment of gene ontogeny and pathway terms linked to immune responses (S7 Fig), particularly genes associated with type I IFN responses (Fig 8E, Table 1). There was no difference in IDO1 expression, a cardinal IFN-γ regulated gene [48] (Table 1). These data suggest lung macrophages in the absence of β6 had increased IFN signaling. To test this further, we measured phosphorylation of IRF3 and STAT1, hallmarks of the type I IFN response, in the alveolar macrophage populations from WT and β6 KO mice. CD11c^+^ CD11b^+^ macrophages from uninfected β6 KO mice had increased phospho-IRF3 and phospho-STAT1 relative to WT CD11c^+^CD11b^-^ AM (Fig 8F).

**Table 1:** Expression of genes associated with type I IFN responses in alveolar macrophages of WT and β6 KO mice.

			Log2 signal			
Probe set ID	Ratio KO/WT (Log2)^a^	Transcript	β6 KO replicate 1^b^	β6 KO replicate 2^b^	WT replicate 1^c^	WT replicate 2^c^
IFN-associated transcription factors						
17497813	4.79	Irf7	11.51	11.37	6.72	6.59
17249593	2.05	Irf1	10.53	10.60	8.58	8.45
17212750	1.95	Stat1	11.37	11.42	9.55	9.32
17300591	1.87	Irf9	9.37	9.28	7.46	7.46
Type I IFN Receptors						
17327069	1.75	Ifnar1	10.49	10.36	8.81	8.54
17327038	1.21	Ifnar2	11.13	10.99	9.97	9.72
IFN-regulated genes						
17249990	2.57	Irgm2	9.80	10.15	7.49	7.32
17385797	2.39	Ifih1	10.84	10.62	8.50	8.19
17269595	2.37	Dhx58	9.77	9.53	7.46	7.10
17423987	1.76	Ddx58	10.36	10.19	8.61	8.42
17450461	4.18	Gbp4	9.50	9.44	5.31	5.27
17403205	3.77	Gbp5	10.19	10.37	6.54	6.48
17450434	3.09	Gbp8	10.54	10.18	7.37	7.16
17450448	2.84	Gbp9	8.90	8.89	6.01	6.11
17450501	2.60	Gbp10	9.96	10.02	7.30	7.49
17403224	1.96	Gbp7	10.19	10.02	8.15	8.14
17403268	3.17	Gbp2	11.31	11.32	8.17	8.12
17403237	2.73	Gbp3	8.92	8.89	6.27	6.09
17403255	1.34	Gbp1	7.11	7.04	5.78	5.70
17450477	0.20	Gbp6	4.17	4.42	4.07	4.11
17358832	4.40	Ifit1	11.28	10.71	6.69	6.50
17358821	-0.10	Ifit3	7.17	7.85	7.54	7.68
17358815	2.75	Ifit2	8.09	7.65	5.26	4.98
17302475	3.77	Irg1	11.75	11.70	7.97	7.94
17411147	3.62	Ifi44	10.97	10.59	7.16	7.16
17219662	4.62	Pyhin1	11.03	10.70	6.27	6.22
17230045	4.27	Ifi204	10.48	10.27	6.54	5.67
17249980	3.39	Igtp	9.68	9.93	6.55	6.28
17230087	2.88	Ifi203	10.46	10.22	7.65	7.28
17230111	2.18	Ifi205	12.68	12.59	10.65	10.27
17531260	1.92	Trex1	8.70	8.64	6.86	6.63
Oligoadenylate synthases						
17452115	5.68	Oas1g	10.56	10.34	4.74	4.79
17452126	2.96	Oas1a	8.33	8.37	5.67	5.11
17441051	4.43	Oasl1	10.93	10.59	6.26	6.39
17452054	4.40	Oas2	11.23	10.93	6.59	6.77
17441037	4.40	Oasl2	11.47	11.41	7.07	7.01
17452070	3.59	Oas3	9.87	9.67	6.15	6.20
IFN-γ-regulated						
17508188	0.55	Ido1	6.7	6.54	6.11	6.04

Sorted alveolar macrophages from WT or β6 KO mice were analyzed by microarray.

^a^Ratio of KO expression to WT expression. Ratio reflects a log2 scale

^b^KO and ^c^WT macrophages were tested in duplicate. Individual fold increase normalized to internal controls.

To determine if type I IFN signaling was also increased in epithelial cells, primary bronchotracheal epithelial cells (mTEC) from WT and β6 KO mice were isolated, differentiated and grown at the air-liquid interface prior to infection with CA/09 virus (MOI 0.1). Phosphorylated and total levels of STAT1 and IRF3 were quantitated at 24 hours post-infection by immunoblot (Fig 8H). β6 KO mTEC exhibited significant increases in total STAT1 and IRF3 levels as compared to WT mTEC after infection as well as at baseline, although differences in phosphorylated STAT1 and IRF3 were minimal (Fig 8H and S6E Fig). Based on these data, we concluded that regulation of type I IFNs was dysregulated in the lungs of β6 KO mice at baseline, and that hematopoietic cells as well as epithelial cells may be involved.

Finally, to determine if this is regulated by TGF-β1, lung homogenates from mice administered exogenous TGF-β1 were monitored for total and phosphorylated STAT1. Treatment decreased phospho-STAT1 in whole lung homogenate (Fig 8I). Macrophages isolated from TGF-β1-treated β6 KO mice also displayed significantly decreased mRNA encoding levels of the IFN associated transcripts Irf7, Ifit1, and Oas1g that were comparable to levels in WT macrophages (Fig 8G) highlighting that the loss of β6 activated TGF-β results in an altered lung macrophage population and increased type I IFN activation. We question whether this was the reason for the enhanced protection from influenza infection.

### Increased survival in β6 KO mice is dependent on type I IFN activity

**Fig 9:**
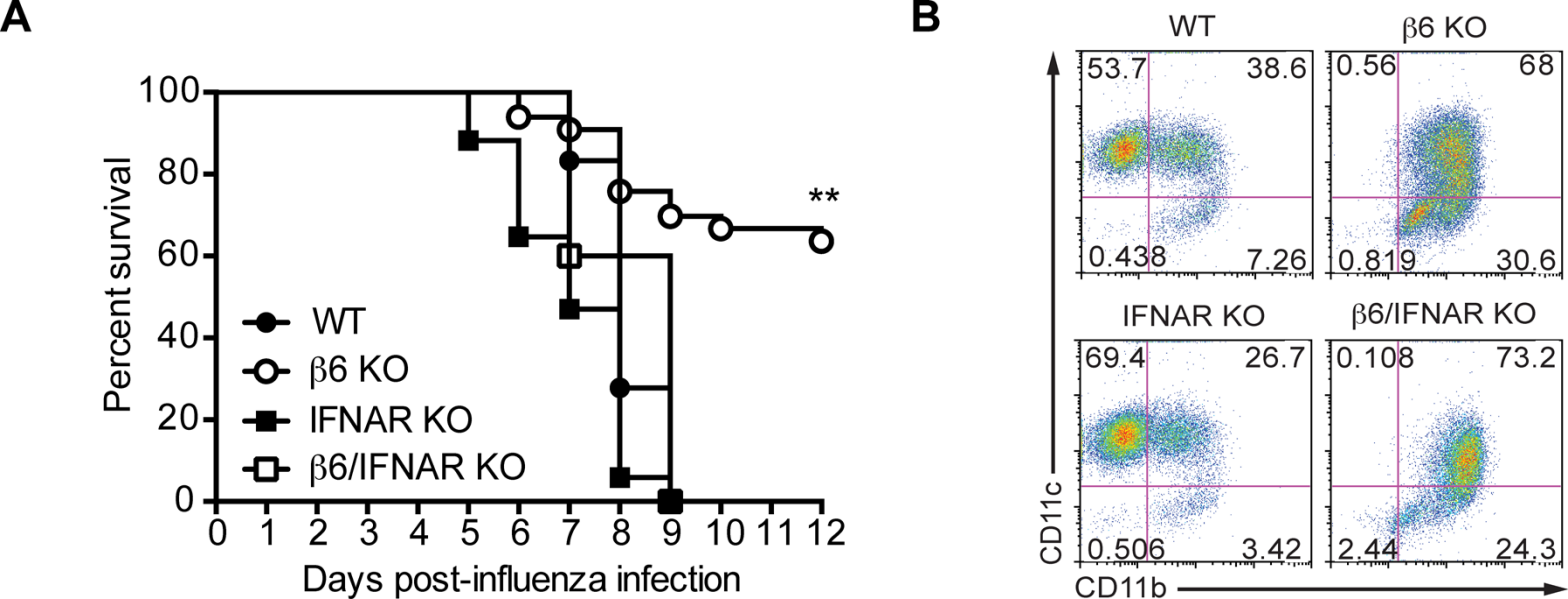
Type I IFN signaling is required for protection of β6 KO mice. (A) WT (littermate controls), β6 KO, IFNAR KO, or β6/IFNAR KO mice were intranasally inoculated with CA/09 H1N1 influenza virus (10^4^ TCID_50_) and monitored for survival for 12 dpi. Data are pooled from 2 independent experiments with n = 5–7 mice per group. p < 0.01 by log rank (Mantel-Cox) test. (B) Alveolar macrophages were analyzed from WT (littermate controls), β6 KO, IFNAR KO, and β6/IFNAR double KO mice.

To determine if the increased type I IFN signaling in β6 KO mice was responsible for the enhanced protection, we crossed β6 KO with *Ifnar* ^-/-^ mice (β6/IFNAR double KO), which lack the type I IFN receptor and all type I IFN signaling, then challenged these mice and their cognate controls created from the same heterozygote crosses with 10^4^ TCID_50_ of CA/09 virus. Mice were monitored for morbidity and mortality for 12 days. β6/IFNAR double KO mice did not survive infection (p = 0.0039 compared to β6 KO, Fig 9A) and exhibited similar mortality to WT mice. Further, imaging WT, β6 KO, IFNAR KO, and β6/IFNAR double KO mice infected with CA/09-NLuc reporter virus at 3 and 7 dpi showed that the ββ6 KO mice had reduced viral spread in the lungs as compared to WT controls (S1A and S1B Fig). In contrast, viral spread in β6/IFNAR double KO mice was comparable to WT and IFNAR KO mice, highlighting the importance of type I IFN signaling in limiting viral spread during infection.

However, the ‘activated’ CD11c^+^CD11b^+^ macrophage phenotype of the β6 KO crosses remained the same as β6/IFNAR double KO (Fig 9B) suggesting the protective anti-viral phenotype of the β6 KO is type I IFNAR-dependent, but another pathway regulates the phenotype of the resident macrophages in the β6 KO independent of type I IFNs.

## Discussion

**Fig 10:**
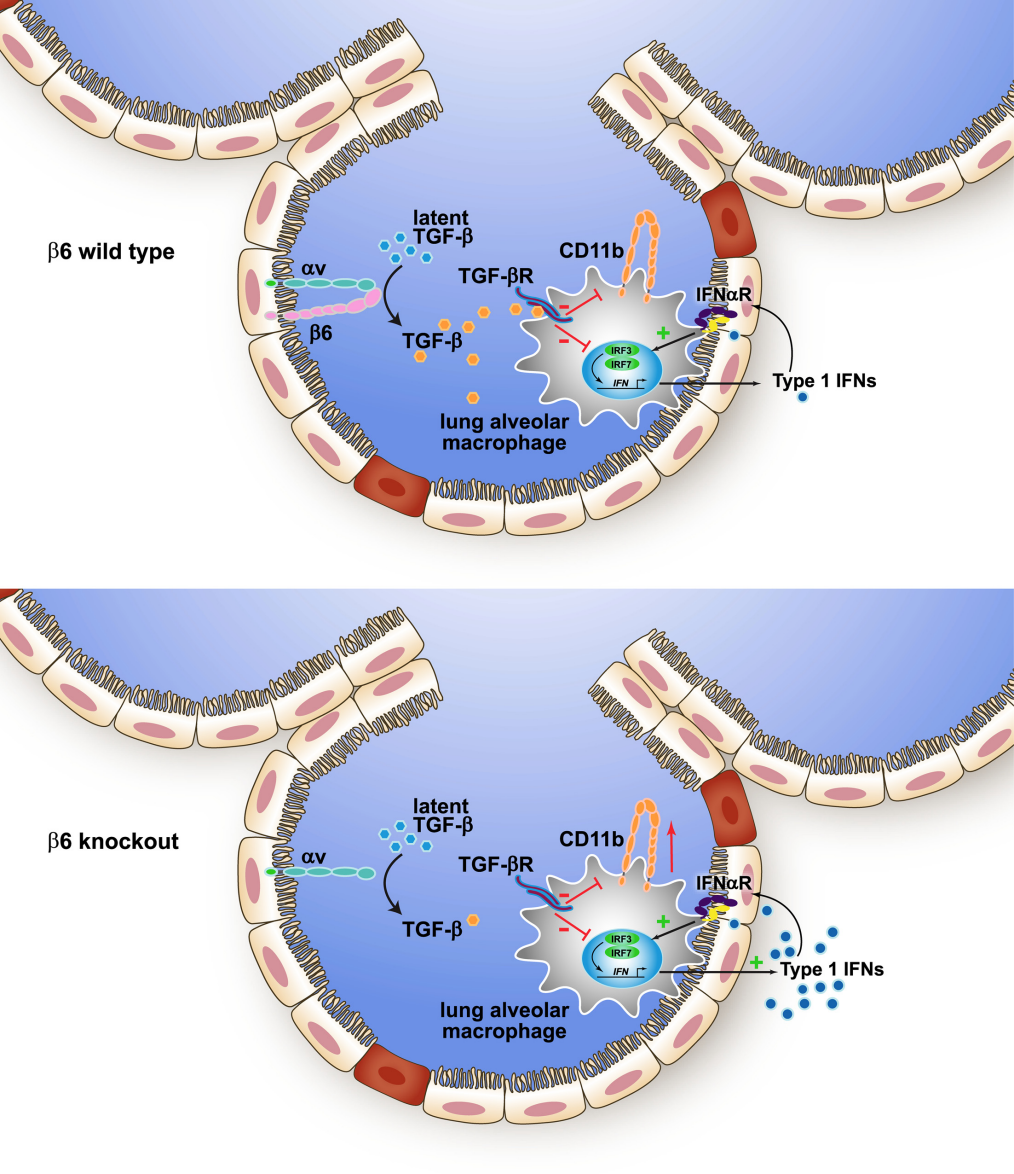
Model for β6 integrin modulation of pulmonary microenvironment. Shown is a depiction of a model whereby TGF-β1, processed by the αVβ6 integrin, directly regulates macrophage cell surface CD11b and the type I IFN response. Other pathways may cooperate with TGF-β1. The exact relationship between the suppression of type I signaling versus the production of type I IFNs through αVβ6 remains to be established.

We propose a model where during pulmonary homeostasis, β6 integrin, possibly by activation of endogenous TGF-β1, suppresses CD11b expression on alveolar macrophages and type I IFN signaling within the lung microenvironment. However, in the absence of β6 integrin, CD11b expression is increased as is type I IFN signaling inducing a ‘primed’ antiviral state in the absence of infection (Fig 10). Through the use of TGF-β1 ‘rescue’ experiments and β6 KO mice lacking type I IFN signaling, we were able to demonstrate the resistance of β6 KO mice to influenza depended on the loss of TGF-β1 and this elevated type I IFN signaling, while the phenotypic changes in lung macrophages were independent of the type I IFN pathway at least at the level of type I IFN signaling as shown in the β6/IFNAR double KO mice.

Overall, our data argue β6 expression leads to changes in the lung microenvironment that are mediated immediately after birth and include alterations in the AM population, and repressed type I IFN signaling in the lung most likely through a major contribution from TGF-β1. This pathway might lead to selective regulation of pathogen colonization or spread, depending on the pathogen’s sensitivity to the pre-existing interferon milieu. Two caveats to the model proposed in Fig 10 include (i) the fact that β6 may regulate factors other than TGF-β1 that cause the downstream effects, and (ii) that we cannot yet definitively distinguish whether β6 negatively regulates type I IFN signaling, type I IFN production or both pathways. In our hands, assays quantitating type I IFN amounts by ELISA or bead-based assays are variable, lacking sensitivity or reproducibility. Furthermore, the potential increase in type I receptor expression on β6 KO macrophages (Table 1) could cause increased uptake of type I IFNs and not accurately reflect the soluble amounts present in the lung at a given time in homeostasis. Thus, the identification of the point at which β6 (with or without TGF-β1) negatively regulates IFN signaling will require new tools and approaches investigating regulation in both macrophages and epithelial cells.

Mice lacking the β6 integrin are protected from disease caused by influenza infection. Compared to WT controls, β6 KO mice develop less severe ALI, as characterized by edema, inflammatory cytokine expression, and vascular permeability of the lung epithelium. β6 KO mice also have altered baseline homeostasis possessing high baseline CD11b^+^ CD11c^+^ macrophages as opposed to CD11b^-^ CD11c^+^ alveolar macrophages. A reasonable interpretation of these data is that β6 KO mice are ‘primed’ for a more efficient response to respiratory pathogens, due to constitutively increased type I IFN signaling at baseline. Importantly, protection extended beyond pandemic H1N1 influenza virus strain to include other influenza virus strains, Sendai virus, and *S*. *pneumoniae* infection. However, protection did not extend to all infections tested. We challenged β6 KO mice with *Francisella tularensis* Schu4 strain using 25 live organisms via the intranasal route. Schu4-infected β6 KO mice had no differences in survival compared to controls consistent with experiments performed in the *Ifnar* ^-/-^ mice [49] (CB, personal communication) (S8A Fig). Although β6 KO mice were significantly protected from disease caused by H7N9 virus (Fig 1D) they were not protected from the highly pathogenic avian influenza (HPAI) A/Hong Kong/483/1997 H5N1 infection (HK/483, S8B Fig). HPAI H5N1 viruses are unique in their ability to rapidly spread beyond the respiratory tract and cause systemic, including neurological, disease [18] and productively replicate in alveolar macrophages [50]. It is possible that HPAI H5N1 viruses are more resistant to type I IFN, that mice succumb due to direct viral cytopathic effect, or complications of systemic infection. Further studies are needed to understand why β6 KO mice are not protected against HPAI H5N1 infection. Taken together, these data suggest β6-mediated modulation of IFN responses affects responses to multiple respiratory pathogens whereas protective responses depend on IFN signaling.

The airway epithelium is a complex barrier comprised of multiple cell types that acts as an interface between the external environment and the internal lung milieu. It serves three main functions in a healthy lung; providing a tight mechanical barrier that rapidly repairs upon insult, mediating innate immune activity to limit foreign antigen invasion, and initiating an inflammatory response through production of cytokines and chemokines [51]. A variety of experimental models and human studies demonstrate the host response to respiratory infection involves initiation, resolution, and restoration phases, all of which must be tightly regulated to prevent disease [52]. However, little is known about how interactions between epithelial cells, which are often the primary targets of respiratory pathogens, and alveolar macrophages, which play a critical role in controlling infections, and the regulation of these responses. Our data identify a pathway by which alveolar epithelial cells normally suppress the anti-microbial activity of alveolar macrophages through a pathway that may involve local activation of TGF-β and subsequent suppression of IFN signaling. In terms of homeostasis, little is known about how type I IFNs control the status of tissue microenvironments. Most work on type I IFN signaling has been associated with anti-viral responses and the regulation of inflammation over the first 18–24 months of life [16]. The implication of our findings is that the lung can tolerate a homeostatic increase in IFN signaling, which provides an advantage against viral spread, but likely comes with ‘costs’ that are not yet clear. We note a recent study has shown homeostatic IFN-β and IFNAR signaling in the brain has an essential role in suppressing neurodegeneration [53]. Therefore, a more comprehensive understanding of the hitherto unknown tissue-specific homeostatic roles of type I IFN signaling is warranted. During infection, although it would seem that upregulation of β6 would only have a negative impact on the host by limiting the anti-viral response, it could also be important for balancing the inflammatory response by suppressing activated macrophages through a TGF-β-dependent process [2,5,15,45], initiating wound repair, or by recruiting macrophages to the site of infection. Attempts to address the role of TGF-β in influenza pathogenesis by systemic inhibition resulted in lethal infection [54]. Thus, future studies will focus on inhibiting the β6 integrin at different times post-infection to understand its precise role in viral infection and its potential as a therapeutic target.

Why is the lung macrophage population different in the β6 KO mice? The αVβ6 integrin is expressed at low amounts in healthy adult tissue but is rapidly upregulated during development and in response to injury and inflammation [30]. Once expressed, β6 activates latent TGF-β1 by binding to the RGD motif in the latency-associated peptide (LAP) [10,55]. Upon activation, TGF-β1 regulates a variety of genes associated with the immune response and pulmonary fibrosis and is thought to negatively restrict inflammation in macrophages through an unknown mechanism [45]. However, systemic inhibition of all TGF-β isoforms by a broadly neutralizing antibody led to lethal influenza infection [54] suggesting that the role of TGF-β during infection may be complex and depend on both the cellular milieu and TGF-β isoform. Recent work has shown that the pulmonary microenvironment dictates the behavior of alveolar macrophages to a greater extent than the origin of the macrophages [42]. Based on our studies we propose that in the lung microenvironment, upregulation of the β6 integrin leads to localized activation of TGF-β1 that negatively regulates alveolar macrophages leading to decreased type I IFN activity in the microenvironment through an as yet undefined mechanism that will require further molecular investigation. An important extension of this model is that influenza viruses themselves activate latent TGF-β during infection [56] and global inhibition of TGF-β during infection is lethal, suggesting an important role in protection from influenza [54]. Therefore, it will be important to be able to experimentally segregate the host- versus virus-specific TGF-β processing events in mice expressing or lacking β6 integrin.

Overall, loss of the β6 integrin resulted in protection from a variety of respiratory infections including influenza and Sendai viruses, bacterial pneumonia, and viral-bacterial co-infections. β6 KO mice had significantly reduced extent and severity of lung injury and inflammation, reduced collagen deposition despite no difference in viral titer, consistent with studies using β6 integrin function blocking antibodies [57], with virus infection being more restricted to airways instead of infiltrating the deep-lung alveolar spaces. β6 function blocking antibodies have been successfully used to improve outcome in idiopathic pulmonary fibrosis and currently recruiting for phase II human clinical trials [58,59]. While the β6 integrin represents an attractive therapeutic target to combat pulmonary disease, it does play a role in lung homeostasis. While transient inhibition of β6 in the lung results in resistance to infection, complete lack of β6 function for long periods of time could be detrimental. Over long periods of time, β6 KO mice over produce macrophage-derived matrix metalloprotease MMP12, leading to emphysema and destruction of pulmonary tissue [16]. However, for short-term protection against the adverse consequences of potentially lethal pulmonary viral infections, our work identifies the αVβ6 integrin as a potentially attractive therapeutic target for transient intervention in lung viral infection.

## Materials and Methods

### Ethics

All animal work was approved by St Jude Children’s Research Hospital Institutional Animal Care and Use committee (protocol #513). *Fransicella* challenges were conducted under protocols approved by the NIAID Rocky Mountain Laboratories Animal Care and Use Committee. St Jude is fully accredited by the Association for the Assessment and Accreditation of Laboratory Animal Care International (AAALAC-I) and has an approved Animal Welfare Assurance Statement with the Office of Laboratory Animal Welfare (A3077-01). These guidelines were established by the Institute of Laboratory Animal Resources and approved by the Governing Board of the U.S. National Research Council.

### Mice

C57BL/6, *Ifnb1*-YFP, CCR2 KO and CD45.1 mice were obtained from Jackson Laboratories (Bar Harbor, ME). *Itgb6* ^-/-^ mice, generated as described [9] and backcrossed 10 generations onto the C57BL/6 background, were obtained from Dean Sheppard (UCSF) then bred at St Jude Children’s Research Hospital. Littermate controls from heterozygous crosses were used as controls. IFNAR KO mice were obtained from Dr. Laura Knoll (University of Wisconsin). Knockouts were confirmed by PCR using primer sets (CCR2, IFNAR) reported on the Jackson Laboratories website, or as previously described (β6) [16].

### Viruses, bacteria and cells

A/California/04/2009 (CA/09) H1N1, A/Puerto Rico/8/34 H1N1, A/Anhui/1/2013 H7N9 and A/Hong Kong/483/1991 H5N1 influenza viruses were propagated in 10-day old specific-pathogen-free embroynated chicken eggs and viral titers determined by tissue culture infectious dose 50 (TCID_50_) on Madin Darby canine kidney cells (MDCK; ATCC CCL-34, Manassas, VA) cells as described [50] and quantitated [60]. Enders strain Sendai virus was propagated as described [61]. D39X *S*. *pneumoniae* was grown in C + Y medium to log phase (O.D. 0.4), pelleted, and diluted to the appropriate concentration in phosphate buffered saline pH 7.4 and stored on ice until infection. MDCK cells were cultured in modified Eagle’s medium (MEM) (Corning, Manassas, VA) supplemented with 2 mM glutamine (Gibco, Grand Island, NY) and 10% fetal bovine serum (Atlanta Biologicals, Lawrenceville, GA) at 37°C, 5% CO_2_.

#### mTEC culture

Primary murine tracheal epithelial cells (mTEC) were isolated as previously described [62]. Briefly, tracheas were collected from euthanized mice and epithelial cells collected by scraping. Cells were cultured on transwell inserts pre-coated with 5 μg/cm^2^ of both rat-tail collagen type I (BD Biosciences, San Diego, CA) and human fibronectin (BD Biosciences). When cells reached 100% confluency, they were cultured at air-liquid interface for 3–4 weeks until fully differentiated (defined as trans-epithelial resistance > 300 Ω cm^2^).

### Infections

#### Influenza virus

Four- to nine-week-old male and female C57BL/6 (WT) mice and β6 KO mice were lightly anesthetized by isoflurane and intranasally inoculated with PBS (control), CA/09 H1N1 (10^4^ TCID_50_), or H7N9 and H5N1 (10^2^ TCID_50_) influenza viruses. Mice were monitored at least daily for morbidity, weights recorded every 48 hours and tissues/BALF collected at different times post-infection. Mice were euthanized if they lost more than 30% of starting weight and exhibited any symptoms of paralysis. H7N9 and H5N1 experiments were performed in a USDA-certified biosafety level 3 enhanced containment laboratory [63].

#### Sendai virus

Mice were deeply anesthetized with Avertin then intranasally inoculated with 2.0x10^4^ plaque forming units (PFU) of Sendai virus (*Enders* strain), monitored daily for weight loss and morbidity. Separate groups of animals were sacrificed at 3d post-infection to collect nasal turbinates, trachea, and lungs for virus titrations.

#### Bacterial challenge

Mice were intranasally inoculated with 4 x 10^7^ colony forming units (CFU) of D39X *S*. *pneumoniae* and monitored daily for morbidity.

#### Secondary bacterial challenge

Mice were anesthetized with isofluorane and intranasally inoculated with 80 TCID_50_ units of PR8 influenza virus, followed by 100 CFU of labeled D39X *Streptococcus pneumoniae* at 7 dpi [32]. Mice were monitored for morbidity, lungs collected at various times post-infection to quantify viral titers, and bled at 48 h post-bacterial challenge (9 d post-influenza infection) for determination of bacterial blood titers.

#### *Fransicella tularensis* challenge

*F*. *tularensis* SchuS4 was propagated as previously described [64] and grown in Mueller Hinton (MMH) broth overnight, aliquoted into 1 ml samples and frozen at -80°C. Immediately prior to use aliquots were thawed and serially diluted to the desired concentration in pharmaceutical grade saline. Inoculum doses were confirmed by plating diluted bacteria on MMH agar and enumerating colonies after 48 hours incubation at 37°C. Mice were anesthetized by a single intraperitoneal injection of 12.5 mg/ml ketamine + 3.8 mg/ml xylazine and immediately inoculated intranasally with 25 CFU *F*. *tularensis* SchuS4. All mice were monitored daily for signs of illness.

#### Bioluminescent influenza infection

WT, IFNAR KO, β6 KO, or β6/IFNAR DKO mice were infected with 10^5^ TCID50 of A/California/04/09 (H1N1) encoding the bioluminescent NLuc reporter on the influenza PA gene (CA/09-NLuc) [34,35]. Briefly, mice were deeply anesthetized with isoflurane before retro-orbital injection of 5 μl of NanoGlo substrate (Promega, Madison, WI) diluted in 95 μl of sterile PBS. Mice were immediately imaged for 3 min using a Xenogen IVIS200 system with LivingImage software (Xenogen, Alameda, CA). Luminescent flux was quantified as photons per second.

### Histopathology and immunofluorescent staining

Deeply anesthetized mice were perfused with 10% neutral buffered formalin, tissues collected at the indicated time post-infection, and embedded in paraffin. H&E and immunohistochemical slides were blinded and scored by PV. Influenza NP staining was performed with anti-NP antibodies (05G, US Biological, Massachusetts, MA) and five separate lung sections quantitated using Aperio ePathology software (Buffalo Grove, IL).

#### YFP staining

Fixed lung tissue from uninfected WT or β6 KO mice containing the *Ifnb1*-YFP reporter was cryoprotected with 20% sucrose prior to embedding. 20 μm thick sections were air dried and subsequently treated with cold acetone for 5 min. Following rehydration in PBS, sections were incubated with Alexa555-conjugated wheat germ agglutinin (1μg/ml; ThermoFisher, W32464) in PBS containing 1% BSA for 30 min at room temperature. Slides were washed in PBS, mounted with hardset media (Vector laboratories), and imaged using a Marianis spinning disk confocal microscope (Intelligent Imaging Innovations) equipped with a 63X 1.2NA objective and EMCCD camera, and analyzed using Slidebook software (Intelligent Imaging Innovations). Ten separate fields were imaged per mouse.

### Quantitative real-time PCR

RNA from whole lung homogenates was isolated using a Trizol (Invitrogen) per manufacturer’s protocol. cDNA was synthesized using the SuperScript VILO cDNA synthesis kit (Invitrogen) and the following parameters: 25°C for 10 min, 42°C for 1 hour, and 85°C for 5 min. PCR was run using 2 μl of cDNA to detect Itgb6, Irf7, Ifit1, and Oas1g mRNA using the Quantitect primer/probe assay system (Qiagen) per manufacturer’s protocol. Samples were normalized to GAPDH mRNA as the internal control.

### Immunoblotting

Whole lungs were collected at different times post-infection and homogenized in RIPA buffer (50 mM TrisHCl pH 7.5, 150 mM NaCl, 0.1% SDS, 0.5% sodium deoxycholate, 1% TritonX-100) containing protease and phosphatase inhibitors (ThermoScientific, Rockford, IL). After centrifugation at 15000 rpm at 4°C, protein concentration was determined using the Bradford assay (Pierce, Rockford, IL) and equivalent amounts loaded onto 10% Tris-glycine SDS-PAGE gels (Lonza, Allendale, NJ). After transfer to nitrocellulose, membranes were probed for integrin β6 (0.236 μg/ml, ch2A1 antibody, a kind gift from Biogen Idec, Cambridge, MA), anti-STAT1 (E-23, Santa Cruz, Dallas, TX, sc-346) (0.1 μg/ml), anti-pSTAT1 (D4A7, Cell Signaling, Danvers, MA, 7649S) (1:1000 dilution), anti-IRF3 (D83B9, Cell Signaling, 4947S) (1:1000 dilution), or anti-pIRF3 (4D4G, Cell Signaling, 4302S) (1:1000 dilution) and detected using HRP conjugated secondary goat anti-rabbit or goat anti-mouse antibodies (0.1–0.2 μg/ml, Jackson) and visualized with ECL. Anti-β-actin (AC-15, Sigma, St Louis, MO, A5441) (1 μg/ml) or anti-GAPDH (mAbcam 9484, Abcam, Cambridge, MA, ab9484) (1 μg/ml) served as loading controls.

### Lung permeability assays

#### Wet lung weight

Lungs were harvested and weighed to determine wet lung weight. Lungs were dried for 96 h at 65°C and weighed again to determine dry lung weight. Lung weight was calculated as (wet lung weight–dry lung weight) to determine weight of liquid in the tissue.

#### Vascular permeability assays

BALF was collected and total protein content quantified by BCA assay (Pierce), and albumin concentration measured by ELISA (Bethyl, Montgomery, TX).

#### Endothelial permeability assay

At 8 dpi, mice were administered 20 mg/kg Evans blue dye (Sigma) by retro-orbital injection. The dye was allowed to circulate for 2 h before mice were sacrificed and perfused with 10 ml PBS. Lungs were harvested and incubated in formamide (Sigma) at 37°C. After 48 h, lungs were removed from formamide and dried at 65°C for an additional 96 h before being weighed. Evans blue dye absorbance was measured by spectrophotometry at 620 nm. The lung permeability index [65] was calculated using the following formula: [absorbance / dry lung weight (g)].

### Analysis of immune cell populations

BALF or whole lung homogenate was collected from PBS control and influenza inoculated mice at 2, 5, 6, or 7 dpi.

#### BALF preparation

Total cell infiltrate into the BALF was counted by Trypan blue exclusion staining.

#### Lung preparation

Lungs were finely minced with a surgical blade and digested in Neurobasal medium (Gibco) containing 1 mg/ml DNaseI (Worthington), 2.5 mg/ml Collagenase P (Roche), 2.5 mg/ml Collagenase/Dispase (Roche), and 1x B27 and N2 supplements (Gibco) at 37°C for 30 min. The digest was dissociated though a 1 ml pipette tip and passed through a 70 μM nylon mesh to obtain a single cell suspension. Cells were treated with Red Blood Cell Lysis buffer (Sigma) prior to further analysis.

#### Staining

Equivalent cell numbers were treated with Fc block (BD Biosciences) and surface stained with combinations of anti-MHC II (M5/114.15.2), anti-CD11c (N418), anti-CD11b (M1/70), and anti-Ly6c (HK1.4) antibodies (eBiosciences) (neutrophils) or anti-Ly6g (1A8), anti-MHC class II (AF6–120.1), anti-CD11c (N418; eBioscience), anti-Ly6c (ER-MP20; AbD Serotech), anti-CD11b (M1/70; BD Biosciences), anti-CD4 (RM4.5; BD Biosciences), anti-NK1.1 (PK136; BD Biosciences), αβT cell receptor (H57–597; BD), anti-CD8 (53–6.7; eBioscience), anti-iNOS (M-19; Santa Cruz Biotechnology), or anti-TNF-α (MP6-XT22; BD Biosciences) (tipDCs, CD4^+^ T cells, CD8^+^ T cells) as previously described [37,66]. CD8^+^ T cells were stained with tetramers specific for the D^b^PB1-F2_62–70_ epitope for 1 h at room temperature prior to surface staining with the monoclonal antibodies as previously described [66].

### Macrophage population analysis

#### Lung digestion

Mouse lungs were finely minced with a surgical blade and digested in Neurobasal medium (Gibco) containing 1 mg/ml DNase I (Worthington), 2.5 mg/ml Collagenase P (Roche), 2.5 mg/ml Collagenase/Dispase (Roche), and 1x B27 and N2 media supplements (Gibco) at 37°C for 30 min. The digest was dissociated though a 1 ml pipette tip and passed through a 70 μM nylon mesh to obtain a single cell suspension. Cells were treated with Red Blood Cell Lysis buffer (Sigma) prior to further analysis.

#### Flow cytometry

Lung cells were treated with Fc block and surface stained with anti-CD11b (M1/70, BioLegend) (1 μg/ml), anti-CD11c (HL3, BD Pharmigen) (1 μg/ml), and anti-F4/80 (BM8, BioLegend) (1 μg/ml) antibodies. The alveolar macrophage population was gated on autofluorescence in the FL-1 channel and high F4/80 expression relative to single color controls. In some experiments further gating with SiglecF (E50-2440, BD Pharmagen, 552126) (1 μg/ml), MerTK (DS5MMER, eBioscience, 12-5751-82) (1:100 dilution), CD64 (X54-5/7.1, BioLegend, 139306) (1:100 dilution), was performed.

#### Microarray analysis

RNA was collected from sorted populations using Trizol (Invitrogen), according to the manufacturer’s protocol. Total RNA (100 ng) was converted to biotin-labeled cDNA (Ambion WTA protocol) and hybridized to a Mouse Gene 2.0 array (Affymetrix) according to the manufacturer’s protocol by the Hartwell Center of St. Jude Children’s Research Hospital. Probe signals from scanned arrays were summarized by the RMA method [67] and Gene Set Enrichment Analysis (GSEA) performed as described [68] using curated pathways from MolSigDB (Broad Institute, Cambridge, MA).

### Adoptive transfers

Sorted populations of macrophages were administered intratracheally to WT or β6 KO mice. For the AM adoptive transfer, autofluorescent FITC^+^ F4/80^+^ lung cells were sorted from donor mice. 200,000 cells were transferred into each recipient mouse in 100 μl PBS.

For transfer of naive PDM, the mouse peritoneal cavity was washed/flushed with 10 ml of PBS, and the F4/80^+^ population sorted. 500,000 cells were transferred into each recipient mouse in 100 μl PBS. After 7 days, mice were sacrificed and AM populations assessed. Donors and recipients were distinguished by CD45.1 and CD45.2 markers.

### Cytokine analysis

BALF or whole lung homogenate was collected from PBS control and influenza inoculated mice at the indicated time post-infection. TNF-α and IL-6 protein expression was determined using the Milliplex Mouse 25-plex Cytokine Detection System mouse cytokine kit (Millipore, Billerica, MA) on a Luminex_100_ 109 reader (Luminex Corp., Austin, TX) according to the manufacturer’s protocol. Cytokine concentration was calculated using a calibration curve obtained in each experiment using the respective recombinant proteins.

### TGF-β ELISA

Active TGF-β1 protein levels were quantified by ELISA assay (BioLegend) on whole lung homogenates according to manufacturer’s protocol.

### TGF-β experiments

Mice were deeply anesthetized with isoflurane and intranasally administered 1 μg of recombinant TGF-β1 (PeproTech, Rocky Hill, NJ) or PBS as previously described [46]. After 18 h, mice were lightly anesthetized and intranasally inoculated with 10^4^ TCID_50_ CA/09 virus. At 2 dpi, mice were given an additional dose of rTGF-β1 or PBS and monitored for morbidity as described. For homeostasis studies, 1 μg of TGF-β1 was administered every 48 hours for 3 weeks.

### Quantitative RT-PCR (IFNs)

RNA from whole lung homogenates was isolated using a Trizol (Invitrogen) per manufacturer’s protocol. cDNA was synthesized using the SuperScript VILO cDNA synthesis kit (Invitrogen) and the following parameters: 25°C for 10 min, 42°C for 1 hour, and 85°C for 5 min. PCR was run using 2 μl of diluted cDNA (1:5 in dH_2_O) to detect IFNa2/IFNa11 or IFNb mRNA using the Taqman primer/probe assay system (Life Technologies) per manufacturer’s protocol. Samples were normalized to GAPDH mRNA expression as the internal control.

### Type I IFN ELISA

IFN-α (PBL Assay Science, Piscataway Township, NJ) and IFN-β (BioLegend, San Diego, CA) protein levels were quantified by ELISA assay on whole lung homogenates according to manufacturer’s protocol.

### Statistics

Statistical analysis was performed with GraphPad Prism (San Diego, CA) as described in the Fig legends. Statistical significance was defined as *p* < 0.05.

### Accession numbers

Microarray data has been deposited in the GEO database with accession number GSE68802.

## Supporting Information

S1 Fig Reduced viral spread in β6 KO mice. (A) WT (littermate controls), β6 KO, IFNAR KO, or β6/IFNAR KO mice were intranasally inoculated with the bioluminescent reporter virus CA/09-Luc (10^5^ TCID_50_). At 3 and 7 dpi, mice were anesthetized and retro-orbitally injected with NanoLuc substrate and imaged (n = 3 mice per group). (B) Quantification of lung flux (photons per second) from imaged mice. Data represents one experiment with n = 3 mice per group. Dotted line = limit of detection.
(TIF)

S2 Fig Gating strategies to identify alveolar macrophages. (A) Flow cytometry gating for lung macrophage isolation. (B) Similar results were obtained whether the macrophages were gated on autofluorescence and F4/80 or autofluorescence and SiglecF.
(TIF)

S3 Fig Alternative gating strategy to identify alveolar macrophages. Similar results were obtained when macrophages were gated using the Immgen method.
(TIF)

S4 Fig Differences in alveolar macrophage phenotype are present at birth and remain unchanged over time. (A) Alveolar macrophages were analyzed from neo-natal WT (littermate controls) and β6 KO mice at the days post-birth indicated. Data are representative of two independent experiments. (B) Alveolar macrophage phenotype is unaffected by raising mice in low dust environments. Pups were born to mothers present in HEPA-filtered cages from the time of earliest detected pregnancy and lines with either AlphaDry bedding or cotton. Lungs were harvested at 14 days post-birth. Data is representative of 2–3 independent litters, n = 4–7 per group. (C) Alveolar macrophages were analyzed from WT (littermate controls), β6 KO, CCR2 KO, and β6/CCR2 double KO mice.
(TIF)

S5 Fig Donor cell recovery from intratracheal macrophage transfers. At 7 days post-transfer, donor cell recovery from the lung was equivalent in the experiments shown in (A) Fig 6B, (B) Fig 6C, and (C) Fig 6E.
(TIF)

S6 Fig Expression of type I IFN in the lungs of WT and β6 KO mice. Whole lung homogenates from mice infected with 10^4^ TCID_50_ of influenza virus were harvested at the indicated time and assayed for IFN-α mRNA (A) and protein levels (B) or IFN-β mRNA (C) and protein levels (D) by qRT-PCR (A, C) or ELISA (B, D). *p < 0.05, ****p < 0.0001 by two-way ANOVA with Bonferroni post-test. Data is representative of 2–3 independent experiments, n = 3–9 per group. (E) Quantification of protein expression shown in Fig 8H was determined using ImageJ software.
(TIF)

S7 Fig Increased type I IFN signaling in macrophages of β6 KO mice. Gene set enrichment analysis was performed on sorted CD11b^-^CD11c^+^ macrophages from WT (littermate controls) lungs and CD11b^+^CD11c^+^ macrophages from β6 KO lungs. Examples of the highest enriched gene sets (FDR < 0.001) are shown to indicate activation of interferon-associated pathways in the β6 KO cells.
(TIF)

S8 Fig Inhibition of β6 integrin is not protective for all respiratory infections. (A) Mice were inoculated with 25 live organisms of *F*. *tularensis* strain Schu4 and monitored for survival. (B) Mice were inoculated with 100 TCID_50_ of A/Hong Kong/483/1997 HPAI H5N1 influenza virus. Data are pooled n = 5–9 animals per group from 2 experiments (A) and n = 6 animals per group from one experiment (B). Survival was not statistically significant by log-rank (Mantel-Cox) test. WT controls were obtained from Jackson.
(TIF)
