# Supplementary figures and images for: An Epithelial Integrin Regulates the Amplitude of Protective Lung Interferon Responses against Multiple Respiratory Pathogens
Source: PLoS Pathog. 2016 Aug 9;12(8):e1005804. doi: 10.1371/journal.ppat.1005804 (PMC4978498; doi:10.1371/journal.ppat.1005804)

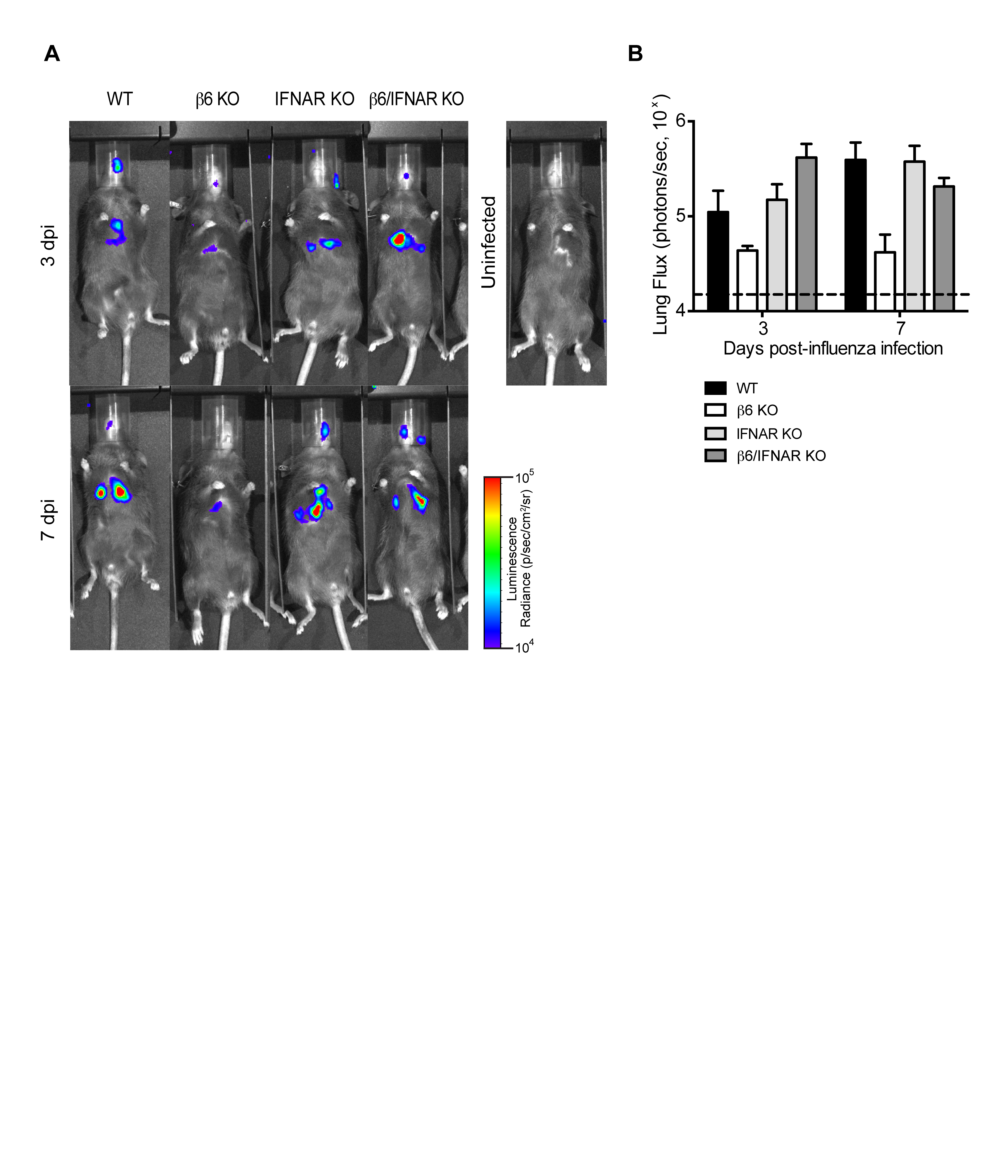

Supplement: S1 Fig — (A) WT (littermate controls), β6 KO, IFNAR KO, or β6/IFNAR KO mice were intranasally inoculated with the bioluminescent reporter virus CA/09-Luc (105 TCID50). At 3 and 7 dpi, mice were anesthetized and retro-orbitally injected with NanoLuc substrate and imaged (n = 3 mice per group). (B) Quantification of lung flux (photons per second) from imaged mice. Data represents one experiment with n = 3 mice per group. Dotted line = limit of detection. (TIF) [file ppat.1005804.s001.tif]

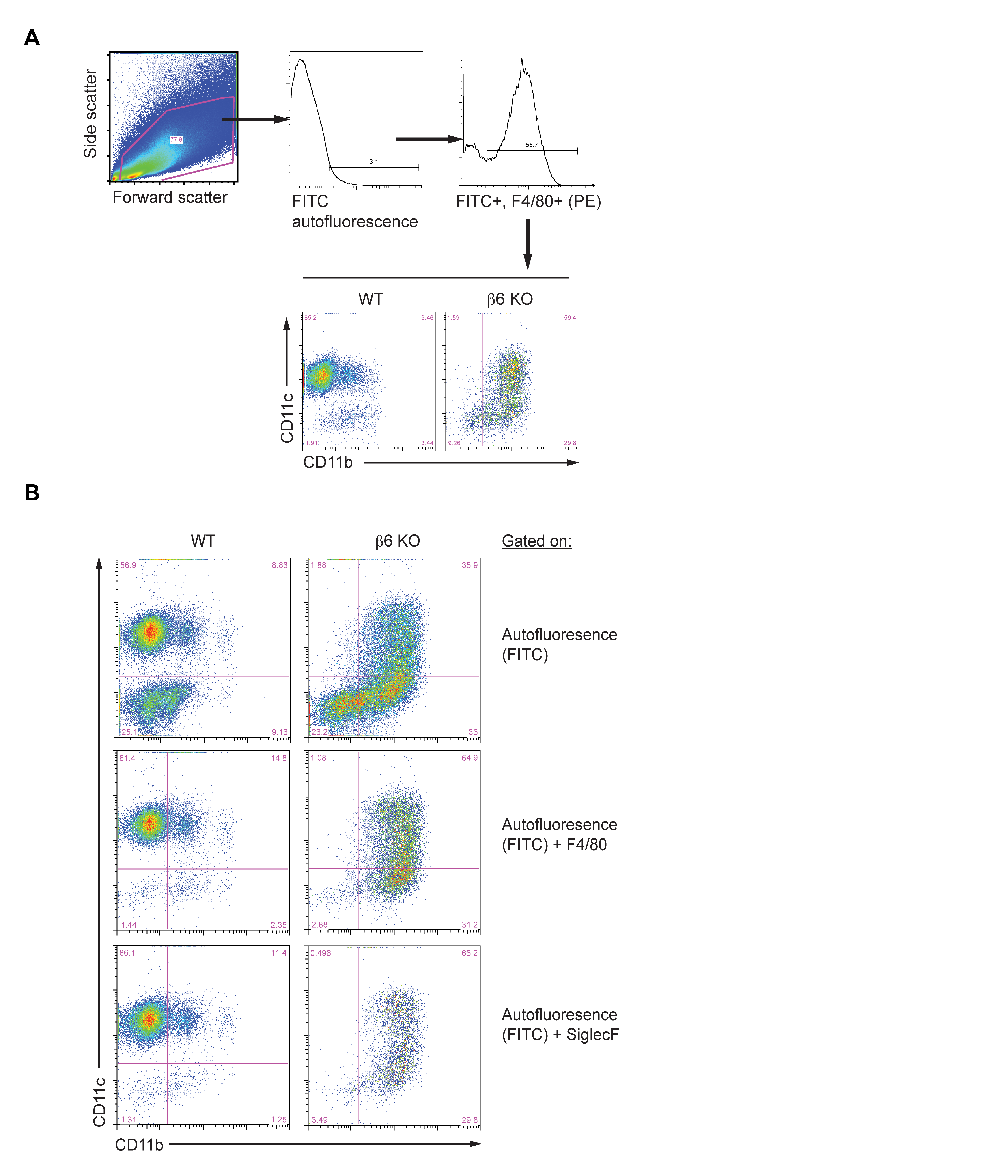

Supplement: S2 Fig — (A) Flow cytometry gating for lung macrophage isolation. (B) Similar results were obtained whether the macrophages were gated on autofluorescence and F4/80 or autofluorescence and SiglecF. (TIF) [file ppat.1005804.s002.tif]

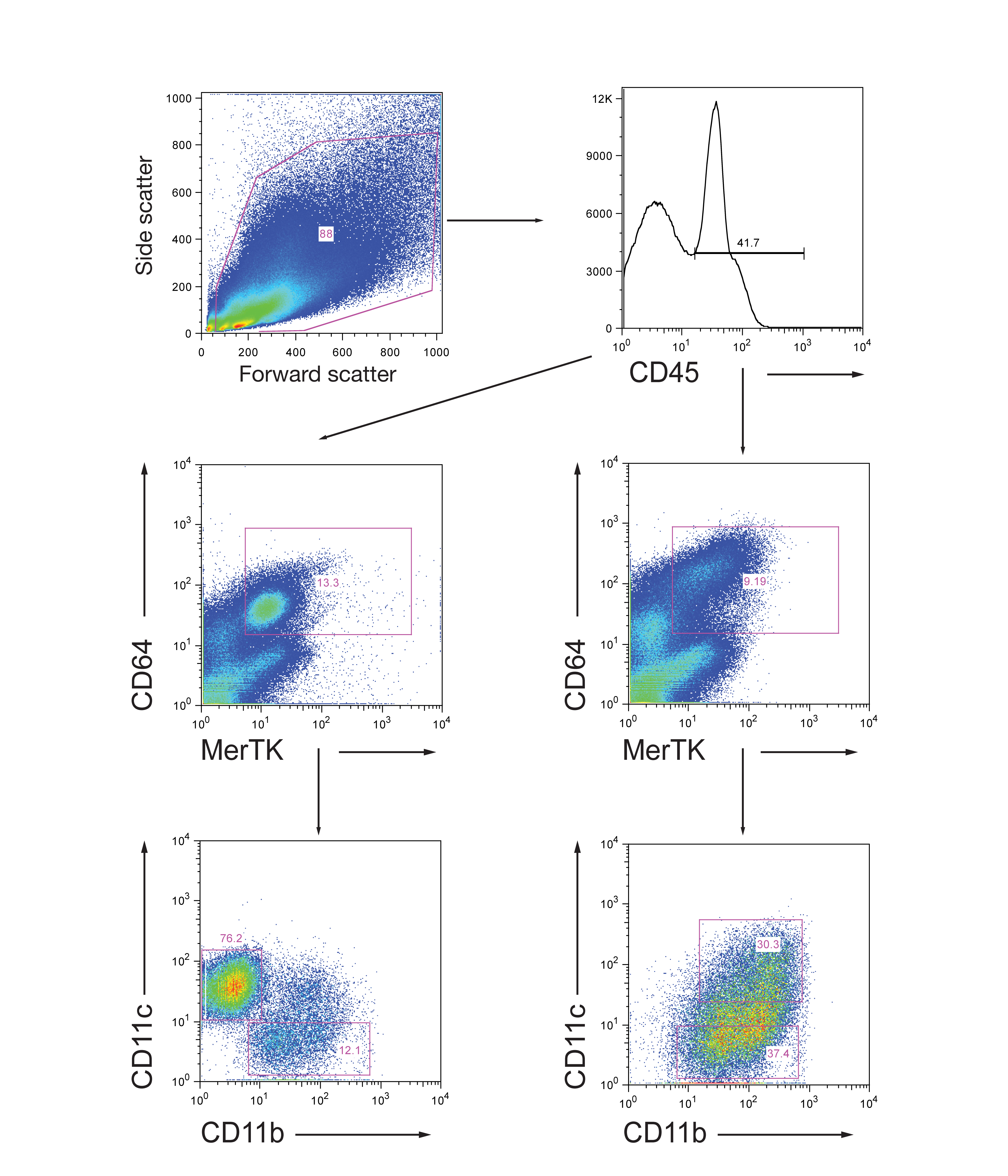

Supplement: S3 Fig — Similar results were obtained when macrophages were gated using the Immgen method. (TIF) [file ppat.1005804.s003.tif]

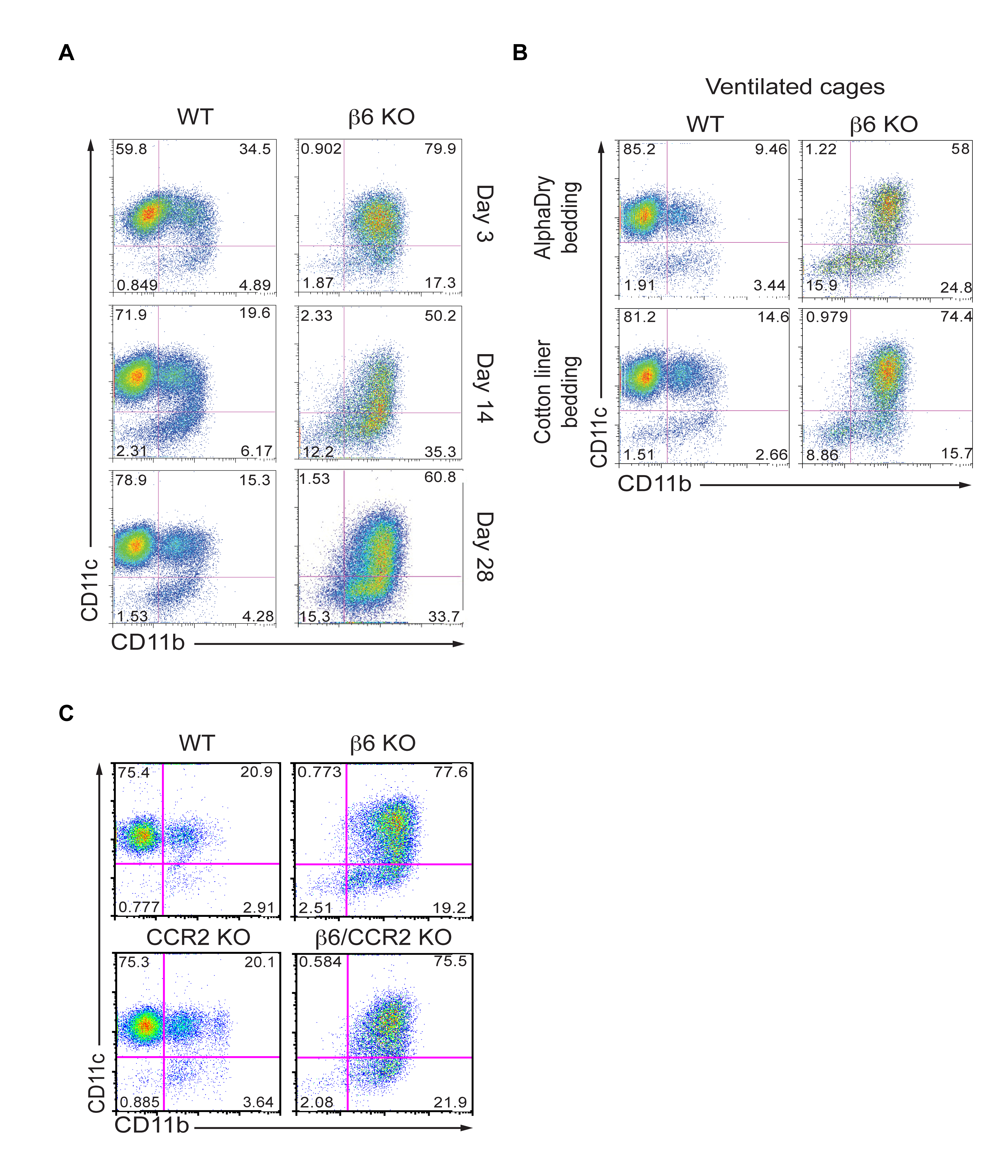

Supplement: S4 Fig — (A) Alveolar macrophages were analyzed from neo-natal WT (littermate controls) and β6 KO mice at the days post-birth indicated. Data are representative of two independent experiments. (B) Alveolar macrophage phenotype is unaffected by raising mice in low dust environments. Pups were born to mothers present in HEPA-filtered cages from the time of earliest detected pregnancy and lines with either AlphaDry bedding or cotton. Lungs were harvested at 14 days post-birth. Data is representative of 2–3 independent litters, n = 4–7 per group. (C) Alveolar macrophages were analyzed from WT (littermate controls), β6 KO, CCR2 KO, and β6/CCR2 double KO mice. (TIF) [file ppat.1005804.s004.tif]

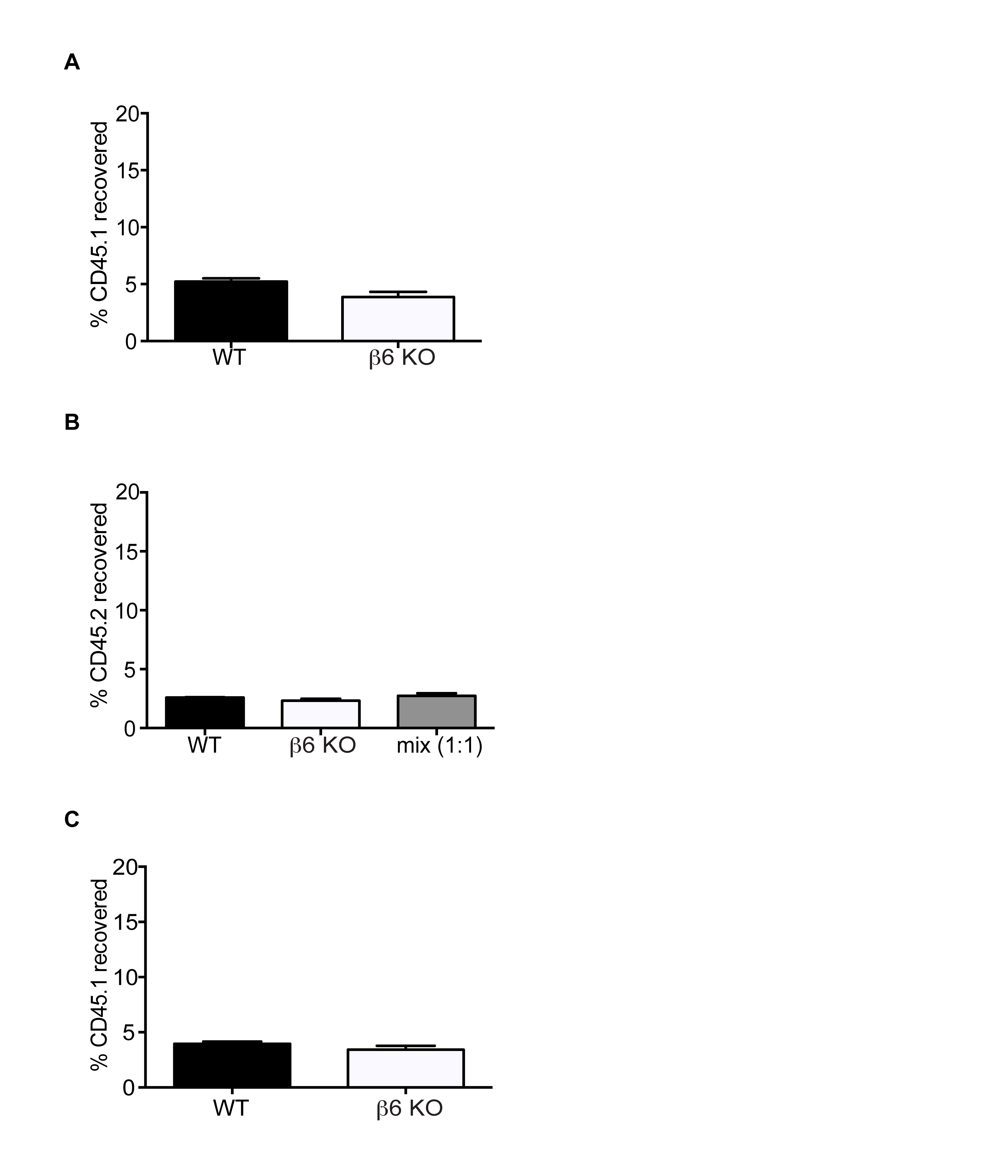

Supplement: S5 Fig — At 7 days post-transfer, donor cell recovery from the lung was equivalent in the experiments shown in (A) Fig 6B, (B) Fig 6C, and (C) Fig 6E. (TIF) [file ppat.1005804.s005.tif]

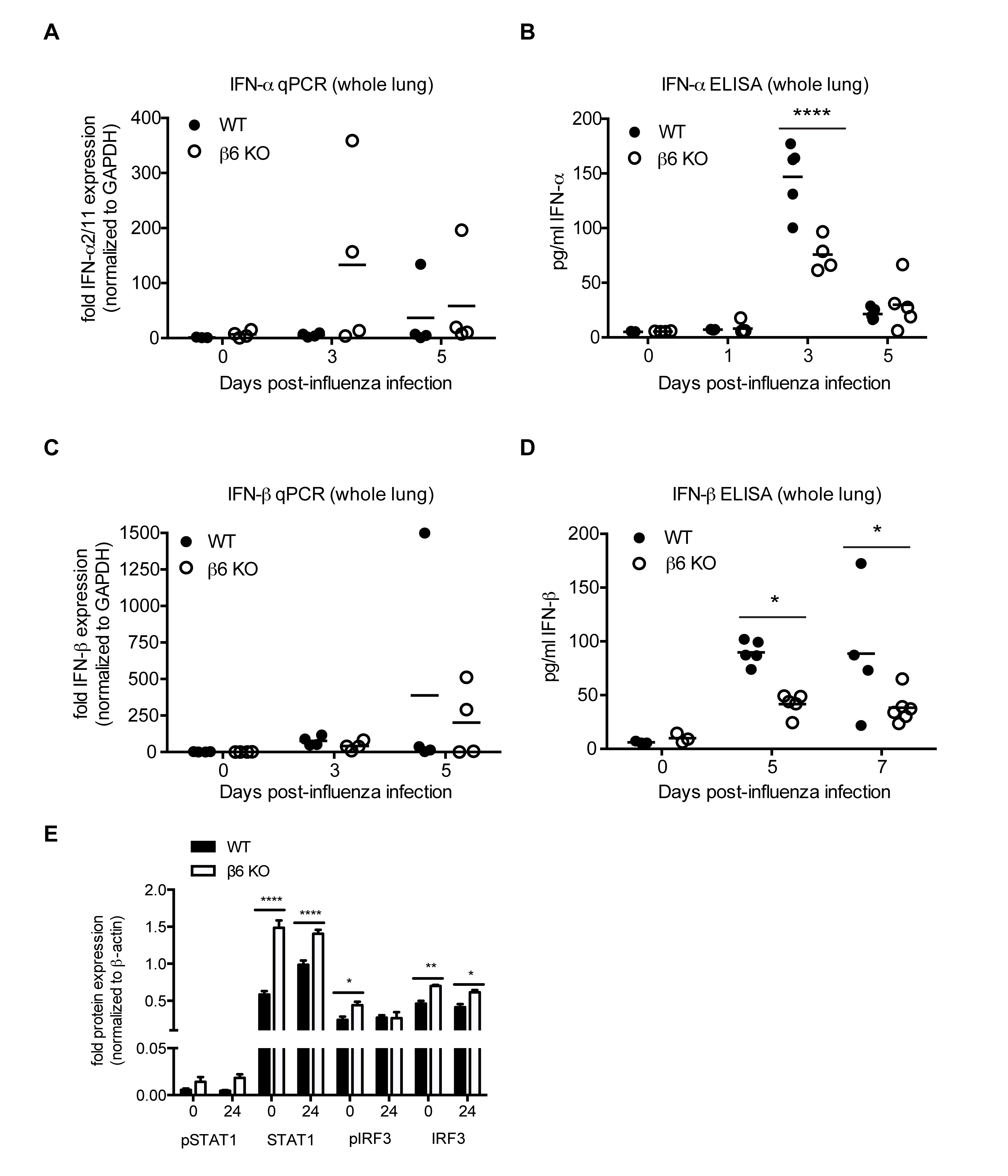

Supplement: S6 Fig — Whole lung homogenates from mice infected with 104 TCID50 of influenza virus were harvested at the indicated time and assayed for IFN-α mRNA (A) and protein levels (B) or IFN-β mRNA (C) and protein levels (D) by qRT-PCR (A, C) or ELISA (B, D). *p < 0.05, ****p < 0.0001 by two-way ANOVA with Bonferroni post-test. Data is representative of 2–3 independent experiments, n = 3–9 per group. (E) Quantification of protein expression shown in Fig 8H was determined using ImageJ software. (TIF) [file ppat.1005804.s006.tif]

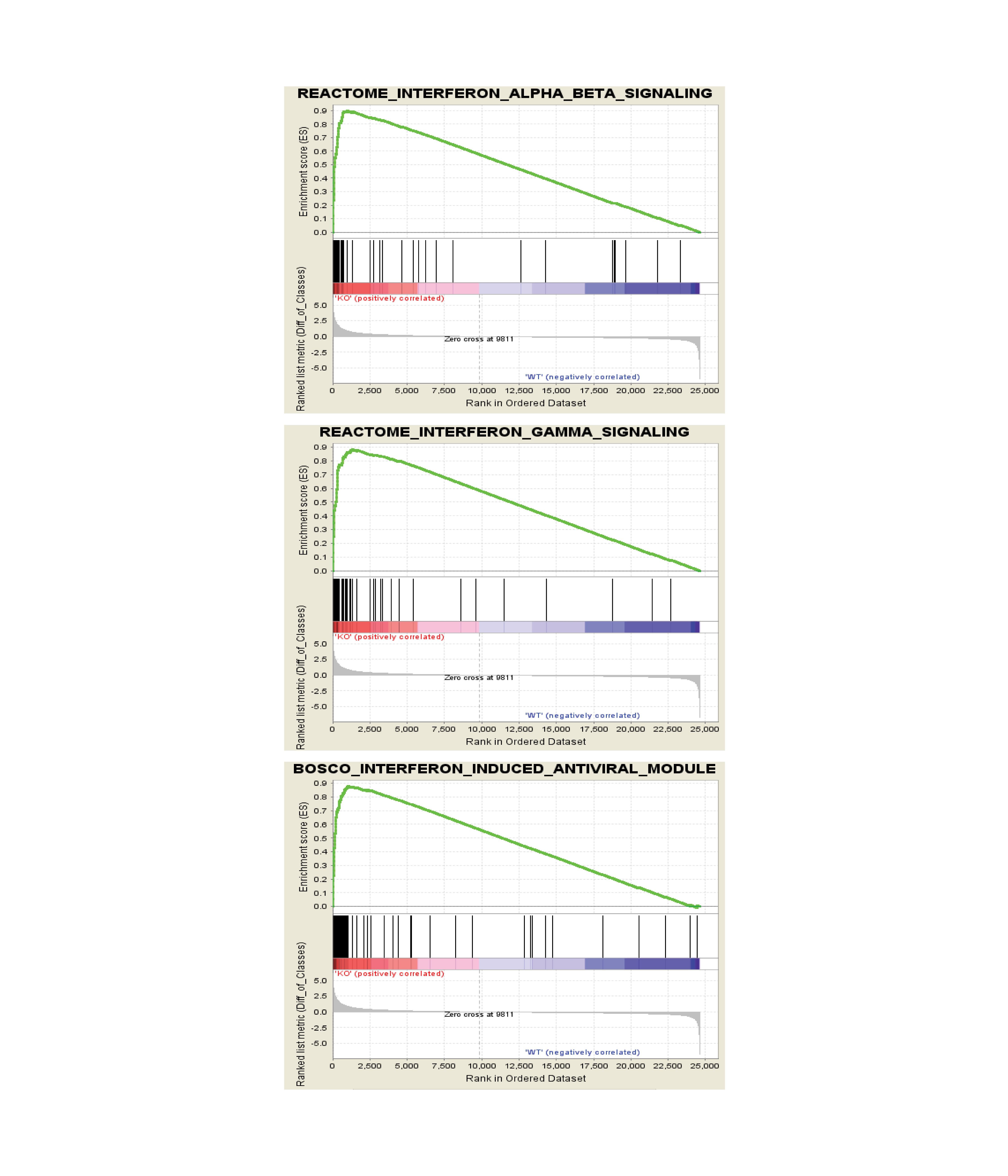

Supplement: S7 Fig — Gene set enrichment analysis was performed on sorted CD11b-CD11c+ macrophages from WT (littermate controls) lungs and CD11b+CD11c+ macrophages from β6 KO lungs. Examples of the highest enriched gene sets (FDR < 0.001) are shown to indicate activation of interferon-associated pathways in the β6 KO cells. (TIF) [file ppat.1005804.s007.tif]

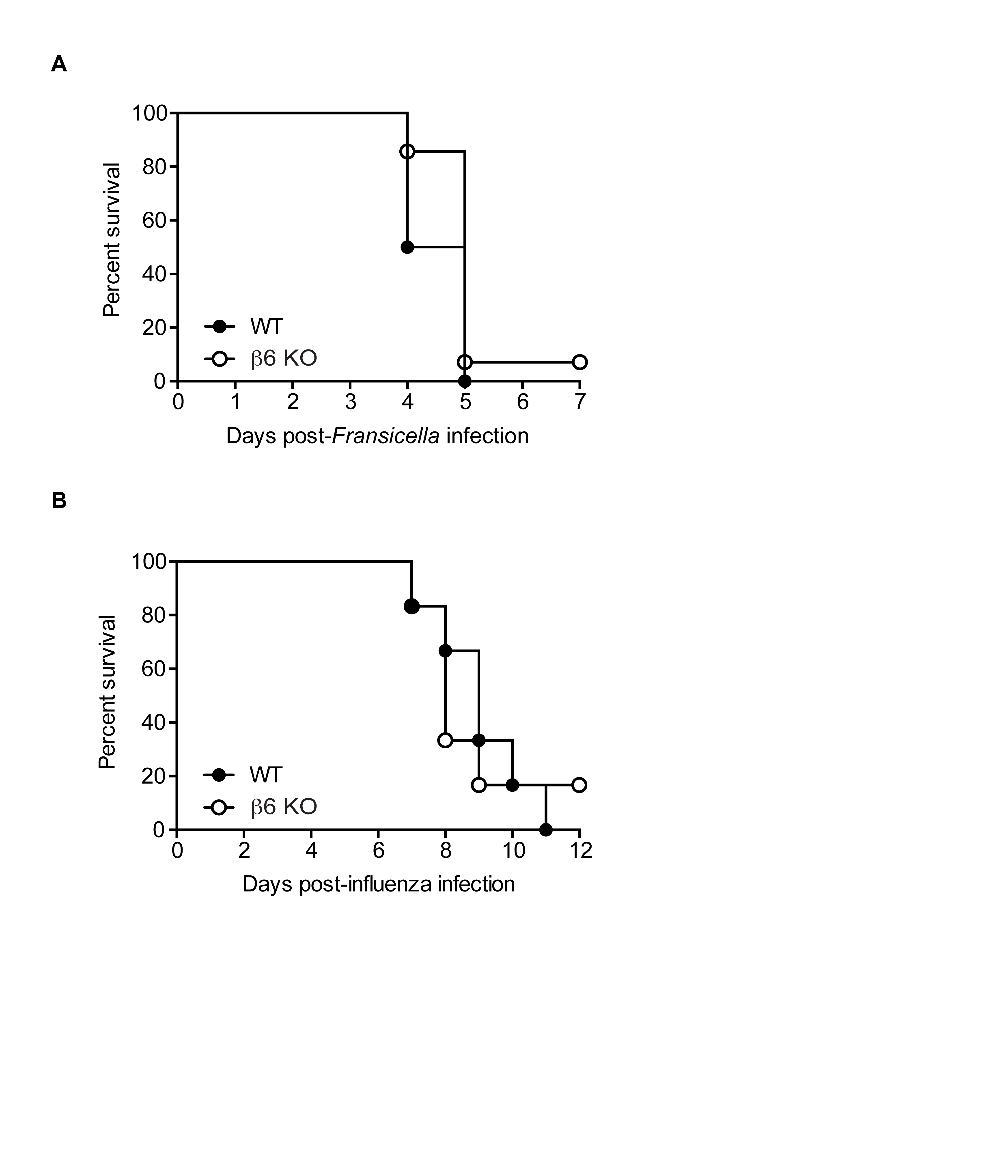

Supplement: S8 Fig — (A) Mice were inoculated with 25 live organisms of F. tularensis strain Schu4 and monitored for survival. (B) Mice were inoculated with 100 TCID50 of A/Hong Kong/483/1997 HPAI H5N1 influenza virus. Data are pooled n = 5–9 animals per group from 2 experiments (A) and n = 6 animals per group from one experiment (B). Survival was not statistically significant by log-rank (Mantel-Cox) test. WT controls were obtained from Jackson. (TIF) [file ppat.1005804.s008.tif]
